# Reductive Catalytic Depolymerization of Semi-industrial Wood-Based Lignin

**DOI:** 10.1021/acs.iecr.1c03154

**Published:** 2021-11-09

**Authors:** Xiaojia Lu, Lucas Lagerquist, Kari Eränen, Jarl Hemming, Patrik Eklund, Lionel Estel, Sébastien Leveneur, Henrik Grénman

**Affiliations:** † Laboratory of Industrial Chemistry and Reaction Engineering, Johan Gadolin Process Chemistry Centre, Åbo Akademi University, Henriksgatan 2, 20500 Turku, Finland; ‡ Normandie Univ, INSA Rouen, UNIROUEN, LSPC, EA4704, 76000 Rouen, France; § Laboratory of Molecular Science and Engineering, Johan Gadolin Process Chemistry Centre, Åbo Akademi University, Henriksgatan 2, 20500 Turku, Finland; ∥ Laboratory of Natural Materials Technology, Johan Gadolin Process Chemistry Centre, Åbo Akademi University, Henriksgatan 2, 20500 Turku, Finland

## Abstract

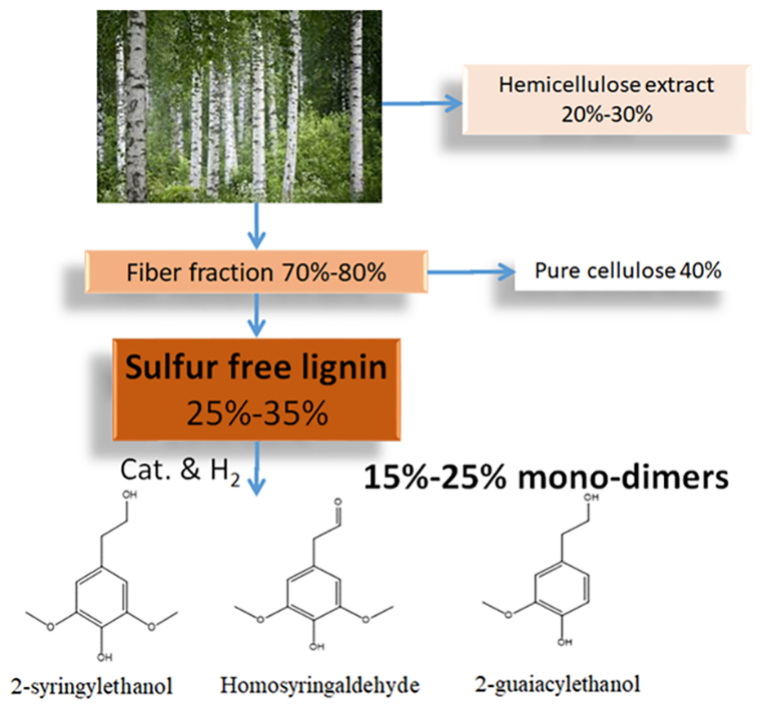

The current work studies the reductive catalytic depolymerization (RCD) of lignin from a novel semi-industrial process. The aim was to obtain aromatic mono-, di-, tri-, and tetramers for further valorization. The substrate and products were characterized by multiple analytical methods, including high pressure size-exclusion chromatography (HPSEC), gas chromatography–mass spectrometry, GC-flame ionization detector (FID), GC-FID/thermal conductivity detector (TCD), and NMR. The RCD was studied by exploring the influence of different parameters, such as lignin solubility, reaction time, hydrogen pressure, reaction temperature, pH, type and loading of the catalyst, as well as type and composition of the organic/aqueous solvent. The results show that an elevated temperature, a redox catalyst, and a hydrogen atmosphere are essential for the depolymerization and stability of the products, while the reaction medium also plays an important role. The highest obtained mono- to tetramers yield was 98% and mono- to dimers yield over 85% in the liquid phase products. The reaction mechanisms influenced the structure of the aliphatic chain in the monomers, but left the phenolic structure along with the methoxy groups largely unaltered. The current work contributes to the development and debottlenecking of the novel and sustainable overall process, which utilizes efficiently all the fractions of wood, in line with the principles of green engineering and chemistry.

## Introduction

**Figure 1:**
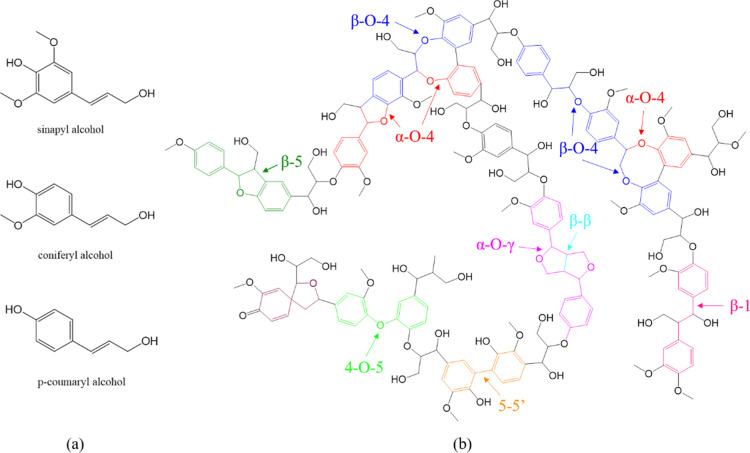
(a) Three phenylpropanol units in lignin; (b) structure and main chemical linkages in lignin.

One of the leading industrial and societal challenges of the 21st century is the shift from the intensive use of fossil resources to renewables in the production of chemicals, materials, and energy. This shift should be performed in a controlled and sustainable way, following the principles of green chemistry and engineering also taking into account the societal and economic aspects. Lignin, which constitutes up to 30% of biomass,^1^ is rich in aromatic polymer components (Figure 1). Lignin extracted from biomass is an essential renewable resource in novel biorefinery applications because it could be used to produce aromatic intermediates and fine chemicals, such as vanillin, phenols, guaiacol, eugenol, and so forth,^2,3^ provided that efficient depolymerization technologies would exist. Lignin is produced in large quantities (>300 billion tons) every year,^4^ however, it has led to limited industrial applications due to its complex and varying molecular structure, broad molecular weight distribution, and variations in the physical–chemical properties.^5^ The extraction of lignin macromolecules from biomass in its reactive, non-condensed form and their efficient depolymerization methods to produce platform chemicals are the major bottlenecks in lignin utilization, as versatile techniques have already been developed for the further valorization of lignin monomers and dimers.^6−10^

Reductive catalytic depolymerization (RCD), in which lignin is processed in an organic solvent (or its water mixture) under a hydrogen atmosphere in the presence of a heterogeneous catalyst, is a promising method for achieving efficient depolymerization.^11−15^ During the process, lignin is depolymerized via catalytic hydrogenolysis while repolymerization is greatly hampered, which is attributed to the reductive stabilization of the reactive intermediates, producing a lignin oil that is rich in monomers and dimers.

The current work focuses on utilizing an industrially modified aqueous extraction based lignin from silver birch, which is currently being produced in pilot scale (500 kg/d).^16,17^ The novel semi-industrial process is relatively green, utilizing mainly water and employing mild soda pulping to efficiently fractionate the hemicellulose and the lignin fraction from cellulose fibers, enabling utilization of the whole biomass for further valorization. This is consistent with the requirements of modern sustainable biorefineries and complies with the principles of green chemistry and engineering. The lignin produced has many advantages over other industrial lignins, such as sulfur-freeness, low carbohydrate content, and relatively high solubility in polar solvents. These characteristics all contribute to the processing of lignin to high-value products.

This work is the first research performed on depolymerization of this novel semi-industrial lignin and it lays the foundation for large scale production of specialty chemicals from this future feedstock produced from an abundant and sustainable wood-based biomass source. Three catalysts, Ru/C (Engelhard, Escat 40), Pd/C (Sigma-Aldrich MKCK3216), and Ni/Al_2_O_3_,^18−20^ were employed to catalyze the depolymerization. Also, several solvents and solvent mixtures were employed to investigate both solubility and reactivity, including water (H_2_O), organic solvent [tetrahydrofuran (THF)], and organic solvent–water mixtures [ethanol–water (EtOH–H_2_O), methanol–water (MeOH–H_2_O), and γ-valerolactone–water (GVL–H_2_O)]. Qualitative and quantitative analytical methods, including high pressure size-exclusion chromatography (HPSEC), GC–MS, GC-FID, GC-FID/TCD, and NMR, were utilized to analyze the substrates and products. Based on the analytical results, the kinetics of the RCD reaction was also studied. The aim of the work was to obtain high yields of monomers and short oligomers for further valorization, while preserving the reactivity in the best possible way by utilizing rather mild conditions.

## Experimental Section

### Chemicals and Materials

Ruthenium on carbon (5%) (Engelhard, Escat 40) was purchased from Engelhard Italiana S.p.A., Italy, in which the moisture content was determined to be 46.75%. Ni/Al_2_O_3_ (5%) was synthesized in the laboratory by a deposition–precipitation method and palladium on carbon (MKCK3216, 5%) was purchased from Sigma Aldrich. All other chemicals were purchased from commercial suppliers and used without further purification.

### Lignin from a Novel Semi-industrial Biorefinery Process

The novel extraction aqueous-based process has been previously described by Von Schoultz.^16,17^ Briefly, modified hot water extraction is first employed to remove the hemicelluloses from birch (betula pendula) chips and then the chips are treated with NaOH to further isolate lignin from cellulose fibers, producing black liquor rich in aromatic polymers.^17^ The lignin is precipitated from the black liquor at pH 2.5 and then washed with acidified water to remove inorganics and water-soluble impurities. The lignin is then collected by filtration and dried. The obtained lignin was shown to have more phenolic hydroxyl groups, carboxylic groups, and less aliphatic hydroxyl groups than milled wood lignin from the same birch chips. The β-O-4 content is relatively low due to the cleavage of the traditionally existing alkyl-aryl ether linkages during the process. Some condensation was also proven to have taken place during the pressurized hot water extraction process by formation of arylglycerols. Nevertheless, this semi-industrial lignin has advantages such as high purity, sulfur-freeness, low carbohydrate content, and a relatively high solubility. Moreover, the high amount of free phenolic hydroxyl groups in the structure are beneficial for modifying the fractions obtained from the depolymerization. A thorough structural analysis of this typical lignin has been described by Lagerquist et al.^21^

### Solubility of the Studied Semi-industrial Lignin

In order to study the solubility of the lignin in the solvent used in analysis, 2 g of it was mixed with 200 mL THF and stirred at 350 rpm at room temperature for 29 h. The mixture was then filtered through a Whatman ashless filter paper (grade 589/2), and the filter paper was weighed before and after the filtration to determine the amount of dissolved lignin. The solubility test was repeated twice, and an average value of solubility was obtained.

The dissolution of lignin during the reaction process was investigated by performing experiments under the same conditions as depolymerization experiments without utilizing catalysts. Predetermined amounts of samples were taken at different stages of the experiment after letting the mixture stabilize for 20 min once the desired temperature was reached. The samples were filtered and dried to determine the amount of dissolved lignin.

### Depolymerization Experiments

**Figure 2:**
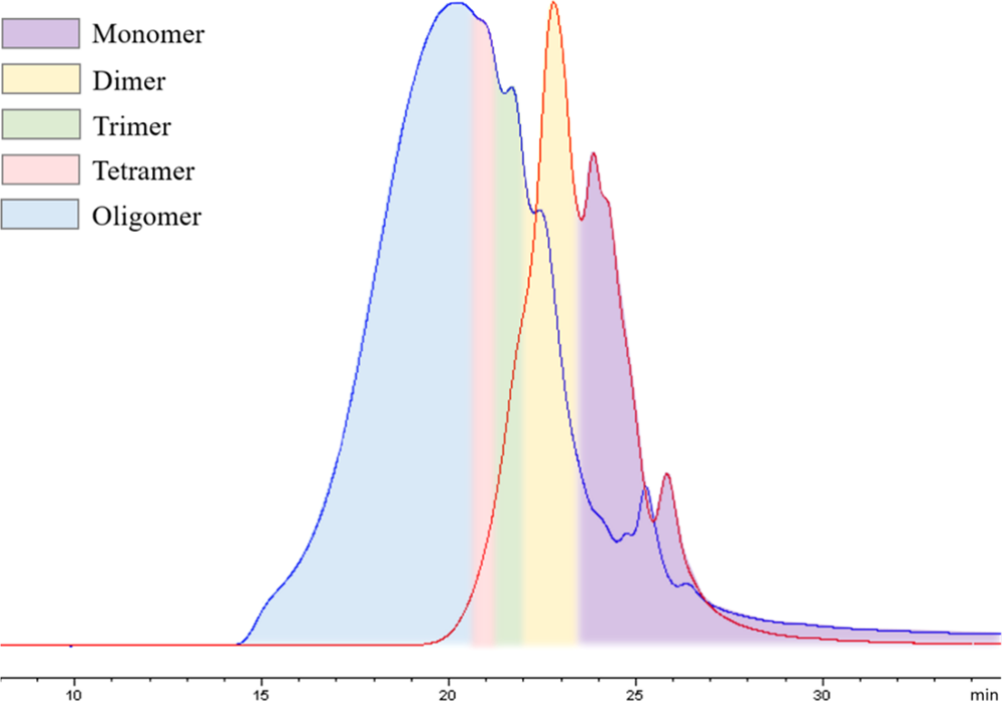
Schematic diagram of semi-quantitative analysis of liquid product distribution by HPSEC, method A. The blue line represents the substrate and the red line, the RCD product.

The reductive catalytic depolymerization of lignin was carried out in a 300 mL Parr reactor with an overhead stirrer. The reactor was equipped with a water/glycol cooling bath, enabling sampling during the reaction by condensing volatile compounds to monitor the changes in the concentrations of substrate and intermediate products with time. Typically, 150 mL of pre-dissolved lignin solution (1 g lignin in 150 mL organic solvent or its aqueous mixture) and a 0.8 g 5% Ru/C (dry) catalyst were charged into the reactor. The reactor was sealed and flushed first with argon and then with hydrogen several times and pressurized to 20 bar with H_2_ at room temperature. The reaction mixture was heated to the desired reaction temperature in less than 30 min; however, high temperatures close to the set point were already achieved earlier. The stirring (1000 rpm) was started after reaching the desired temperature, which was defined as time zero. The experiment lasted for 24 h and liquid samples (3–4 mL) were taken at regular time intervals to monitor the progress of the reaction. The end of the sampling tube was placed below the meniscus and a sinter was installed to avoid the loss of catalyst while taking samples. The intermediate samples withdrawn through the water/glycol cooling bath were filtered with a 0.45 μm poly(tetrafluoroethylene) (PTFE) filter to remove the catalyst and possible undissolved lignin. The samples were then dried and dissolved in THF in a concentration of 1 mg/mL for HPSEC analysis. After a 24 h experiment, the reactor was cooled to room temperature. After cooling, the gas product was collected through a sampler connected to the vent on the lid of the reactor and directly subjected to GC-FID/TCD analysis before the reactor was carefully depressurized. The liquid product was filtered and rinsed with additional solvent. The filtrate was collected, and the solvent was removed by rotary evaporation, as preparation for further analysis. In experiments aimed at studying mass balance, samples were not taken during the reaction, and only the final oil products and gaseous samples were collected. The composition of the lignin oil was also semi-quantitatively analyzed by HPSEC, as illustrated in Figure 2. The molar masses of lignin oil products are between 100–700 g/mol, which corresponds to DP lower than 4. The results were also supported by LC/MS analysis, as shown in Figure S1.

The mass balance was calculated as follows
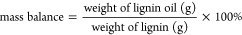
1

### Analysis of Gaseous Products

The composition of gaseous products collected at the end of the experiment was determined by a gas chromatograph equipped with a J&W GS-Q PLOT column (30 m × 0.53 mm). The front detector was a flame ionization detector (FID), utilized mainly for determining hydrocarbons, while the back detector was a thermal conductivity detector (TCD) for inorganic gas products. The peaks were identified by GC–MS.

### Ultrafiltration

After selected experiments, the reaction mixture was transferred to a Millipore ultrafiltration system equipped with a regenerated cellulose membrane. A membrane with a pore size of 1 kDa was utilized in the current work. The solvent resistant stirred cell was sealed and pressurized to 4.75 bar with nitrogen after which the stirring was commenced and the speed was set to 265 rpm. The ultrafiltration was performed at room temperature. After the ultrafiltration, the filtrate was collected and dried for analysis. The residue, which contained large molecular products and the catalyst, was diluted to 150 mL using the same solvent as in the RCD experiment after which, it was introduced to a further oxidation experiment under 5 bar O_2_ and 240 °C for 24 h.

### Chemical Characterization of Lignin Oil Products

#### Molecular Weight Distribution

The molecular weight profile of the lignin substrate and in the liquid phase samples was determined by HPSEC utilizing two different equipment and methods. Either a (A) Agilent 1100 Series high-performance liquid chromatography (HPLC) instrument equipped with a G1315B DAD-detector or a (B) Shimadzu HPLC instrument equipped with an LT-ELSD detector was employed. 2 × Jordi Gel DVB 500 A (300 mm × 7.8 mm) columns (Columnex LLC, New York, NY, USA; 40 °C) + guard column (50 mm × 7.8 mm) in series were utilized in both systems. The analysis was carried out at 40 °C with one percent acetic acid in THF as the eluent at a flow rate of 0.8 mL·min^–1^ with 35 min analysis time/sample. The dry samples were dissolved in THF to yield a concentration of 1 mg/mL. The samples were then vortexed for 0.5–1 min and filtered with a 0.45 μm PTFE filter to remove any insoluble particles before injection. The product distribution was semi-quantitatively analyzed by HPSEC, method A, as accurate quantitative analysis of lignin monomers and dimers is still a considerable challenge even with modern technology. The calibration was performed with polyethylene standards in a wide range of molecular weights while syringaldehyde and hydroxymatairesinol were used as low molecular weight standards, as shown in Figure S2, based on which the peaks of monomers, dimers, trimers, and tetramers could be approximately quantified based on the retention times, as illustrated in Figure 2. One sample was also directly injected to an ion-trap MS system (Figure S1) to compare with the molar masses of the low molar mass compounds observed in the HPSEC analysis.

#### Quantitative GC Analysis

The composition of lignin oil products was quantitatively analyzed using gas chromatography equipped with an FID detector, auto-sampler, and Agilent J&W HP-1/SIMDIST column of dimension 6–7 m (L) × 0.530 mm (ID), film thickness of 0.15 μm. The carrier gas was H_2_ and the injection volume was 0.5 μL. Initial injection temperature was 80 °C (0.1 min) with a temperature rise of 50 °C/min up to 110 °C and then at 15 °C/min to the final temperature of 330 °C (7 min). The initial oven temperature was 100 °C (0.5 min) with a temperature rise of 12 °C/min up to 340 °C (5 min), also the detector temperature was 340 °C. For the quantitative analysis, internal standard (kolesterol, 0.02 mg/mL) was added before silylation by a mixture of (pyridine: N,O-bis(trimethylsilyl)trifluoroacetamide: chlorotrimethylsilane = 1: 4: 1). The peaks of lignin derived compounds were identified by GC–MS by comparing with an in house spectral database.

#### NMR Spectroscopy

All the NMR experiments were performed at 25 °C in DMSO-*d*_6_ on an AVANCE III spectrometer (Bruker Biospin GmbH, Rheinstetten, Germany) operating at 500.13 MHz for ^1^H and 125.77 MHz for ^13^C and 202.46 MHz for ^31^P. HSQC experiments used Bruker’s pulse program “hsqcedetgpsisp2.3” for multiplicity edited with a spectral width of 8012 Hz (from 3.3–12.7 ppm) and 20 750 Hz (from 7.5–157.5 ppm) for the ^1^H- and ^13^C-dimensions. The residual solvent peak was used as the internal reference δ_H_/δ_C_ (2.50/39.52 ppm). A common standard protocol was utilized for ^31^P NMR sample preparation.^22^

## Results and Discussion

### Solubility of the Lignin

The solubility of lignin in organic solvents is not easily predictable, as it is determined by many factors such as chemical structure, molecular weight, and the presence of hydrophilic moieties in the lignin molecule.^23^ Therefore, the solubility data of other lignins are basically of no reference value for the current study.

We tested the solubility of lignin in THF at room temperature to have a basic understanding of the thermodynamic properties of this novel semi-industrial wood-based lignin. The average value of solubility of the lignin in THF was 8.76 mg/mL. It is relatively high compared to lignin from other sources. For instance, the solubility of kraft lignin in THF was reported to be 1.44 mg/mL.^24^ Lignin solubility decreases with condensation, which at least partly explains the difference.

The dissolution of 1 g lignin in the reaction medium [150 mL EtOH–H_2_O (50/50, v/v)] under reaction conditions (240 °C) was also determined. It is evident from the results presented in Figure S3 that the concentration of dissolved lignin increased with temperature and that the lignin was completely dissolved in the EtOH–H_2_O mixture at reaction temperature (240 °C). The concentration slightly increased with time under isothermal condition, which could partly be due to the loss of solvent to the gas phase. Correspondingly, the concentration of dissolved lignin decreased while the reaction mixture was being cooled down. The relatively low concentration observed at the same temperature during the cooling process when compared with the heating process may be caused by repolymerization of lignin fragment in a catalyst-free environment.

### Study of Different Factors Influencing the Results

#### Catalyst Screening

**Figure 3:**
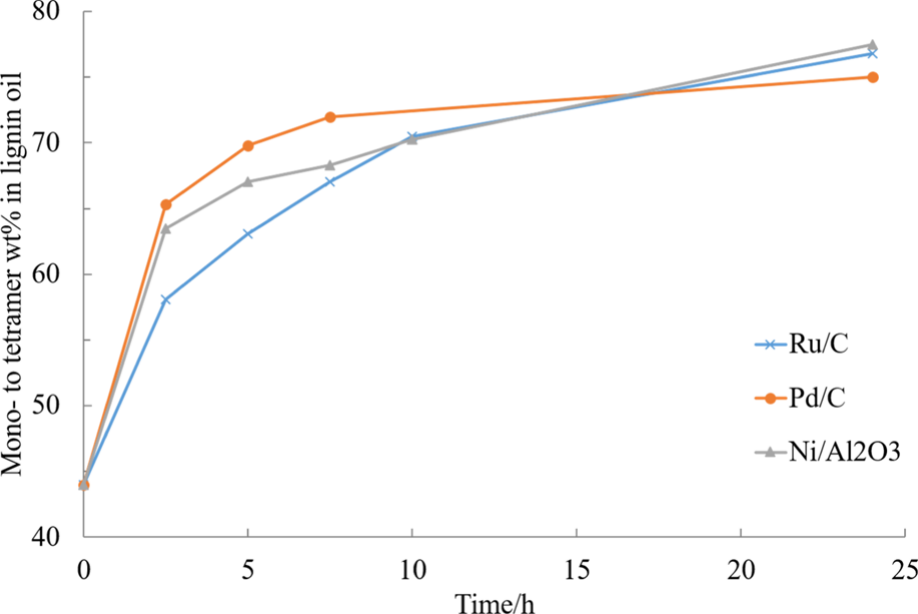
Effect of catalyst type on the mono- to tetramer composition in experiments performed in EtOH–H_2_O (50/50, v/v) under 20 bar H_2_ and 240 °C.

Three different heterogeneous catalysts (Ru/C, Pd/C, and Ni/Al_2_O_3_) were tested in the current work by adding an equivalent amount of each (dry basis) to the reaction mixture while maintaining the reaction conditions identical. The reaction catalyzed by 5% Ru/C produced less of the polymeric fraction, showing a good conversion efficiency towards smaller aromatic compounds (mono- and dimers). Palladium on carbon displayed faster kinetics compared to Ru/C, however, ethane, propane, and butane were observed in significant amounts in the analysis of the gas phase showing significant unwanted cleavage of the aliphatic part of the lignin compounds.^25^ It was observed that RCD reaction with Ru/C was slower but the final yield, exceeded the one obtained with the other two catalysts after 24 h experiments. Ni/Al_2_O_3_ performed also rather well, however, Ru/C was chosen due to the low acidity and good performance. The final mono- to tetramer yield (weight percentage in lignin oil products) was calculated to be about 77% for the Ru/C catalyzed experiment, while it was 75% for Pd/C, as displayed in Figure 3.

#### Effect of Sampling and Lignin Dissolution Kinetics

In the current study, we installed a water/glycol cooling bath to condense gaseous compounds during sampling, when investigating the evolution of the RCD reaction. To study if the sampling during the experiments influenced the lignin oil composition due to volatiles formation, experiments with and without sampling during the experiment were conducted under identical conditions and the results are shown in Figure S4. The results confirmed that sampling during the experiment did not influence the results obtained in the liquid phase.

The effect of loading the lignin as a solid or pre-dissolving it 24 h prior to the experiment was studied in order to see if the solid–liquid dissolution rate influenced the depolymerization rate. The experiments were carried out under the same reaction conditions, and the loadings of lignin and solvent were identical. The results shown in Figure S5 confirmed that the dissolution kinetics did not influence the depolymerization kinetics.

#### Effect of the Presence of Only H_2_ or the Catalyst

**Figure 4:**
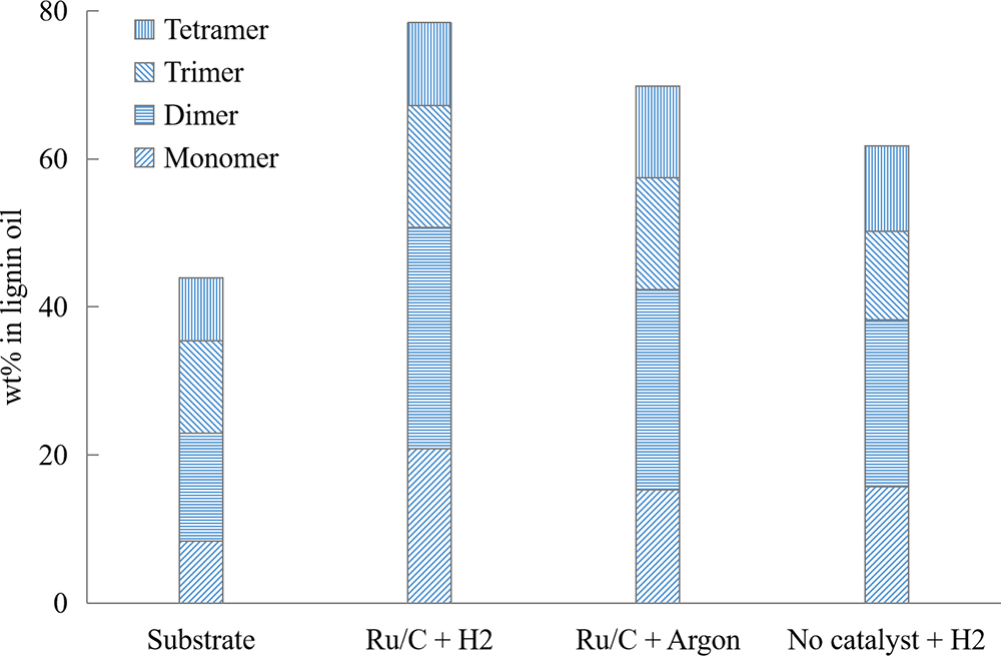
Effect of the presence of H_2_ and catalyst on the mono- to tetramer composition after a 24 h experiment in EtOH–H_2_O (50/50, v/v) mixture at 240 °C compared to a blank experiment with only substrate.

The influence of H_2_ and the presence of catalyst were studied and compared to reference experiments (blank and only substrate), as shown in Figure 4. The final samples from experiments performed without one of these two elements were observed to be much more condensed than the sample from a typical RCD reaction. The absence of a catalyst seemed to have a more significant impact on the final product compared to the absence of H_2_. An explanation might be that the solvent acted as a hydrogen donor in the reaction conducted under an argon atmosphere.^1,26^ However, a significant difference can still be noticed between the final product of the non-catalytic experiment under H_2_ atmosphere and the substrate, which implies the occurrence of non-catalytic reactions and/or thermal degradation (TD). This was confirmed by a mono- to tetramer yield of 62%.

#### Kinetics of the Depolymerization

**Figure 5:**
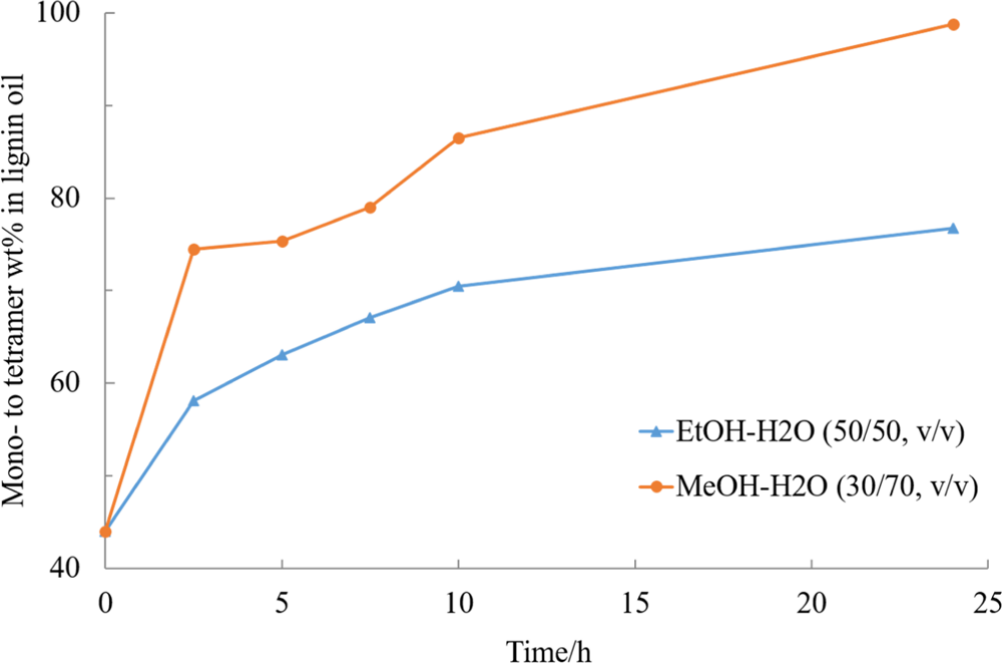
Effect of reaction time on the mono- to tetramer composition of experiments in MeOH–H_2_O (30/70, v/v) and EtOH–H_2_O (50/50, v/v) mixtures catalyzed by 5% Ru/C under 20 bar H_2_ and 240 °C.

The water/glycol cooling bath connected to the reactor enabled reliable sampling for studying the kinetics. The samples of two experiments conducted in MeOH–H_2_O (30/70, v/v) and EtOH–H_2_O (50/50, v/v), respectively, were analyzed by different methods. It is evident from the results presented in Figure 5 that low molecular weight compounds were formed with time, whereas the proportion of polymers in the product mixture decreased. The lignin mono- to tetramer fraction constituted 77% of the products in a EtOH–H_2_O mixture and 98% in MeOH–H_2_O mixture after 24 h of reaction in the at 240 °C with the Ru/C catalyst and 20 bar hydrogen, which demonstrated that the lignin polymer was efficiently cleaved into smaller compounds as the experiments proceeded. Smaller products than aromatic monomers were not detected in the liquid phase, which shows that the aromatic lignin monomer basic units were not degraded during the experiments.

**Figure 6:**
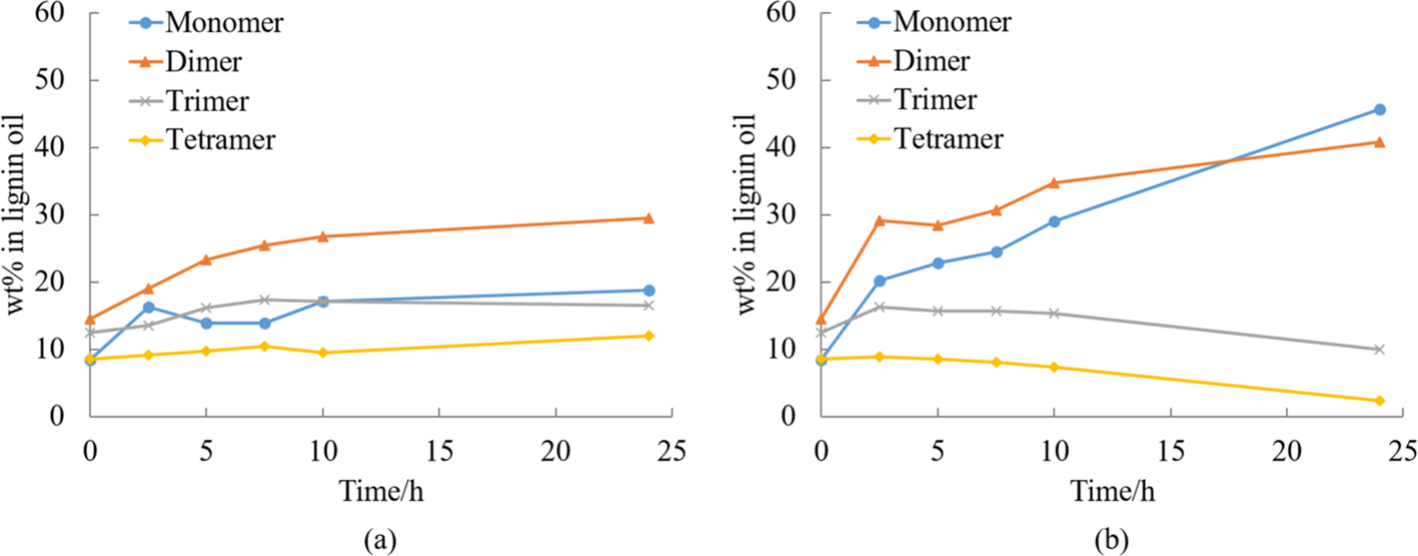
Effect of reaction time on the composition of mono-, di-, tri-, and tetramer of experiments in (a) EtOH–H_2_O (50/50, v/v) and (b) MeOH–H_2_O (30/70, v/v) mixtures catalyzed by 5% Ru/C under 20 bar H_2_ and 240 °C.

Figure 6 displays the concentration of the mono, di, tri, and tetramers as a function of time. When comparing with the results in Figure 5, it is evident that consecutive reactions occurred. It can be seen in Figure 5 that the total amount of mono- to tetramers increases with time, indicating the depolymerization of lignin macromolecules. Results in Figure 6, however, show that the concentrations of tri- and tetramers decrease while those of mono- and dimers increase. It can be concluded that the polymer was first cleaved to oligomers, which then further reacted to form dimers and monomers. This is especially evident in Figure 6b, where the cleavage of the oligomers to dimers and monomers was observed to be more efficient when methanol water mixture was used instead of ethanol water mixture. This is most probably attributed to the higher polarity of the solvent, as discussed in more detail in section “Effect of solvent”.

#### Effect of Hydrogen Pressure, Temperature, and pH

**Figure 7:**
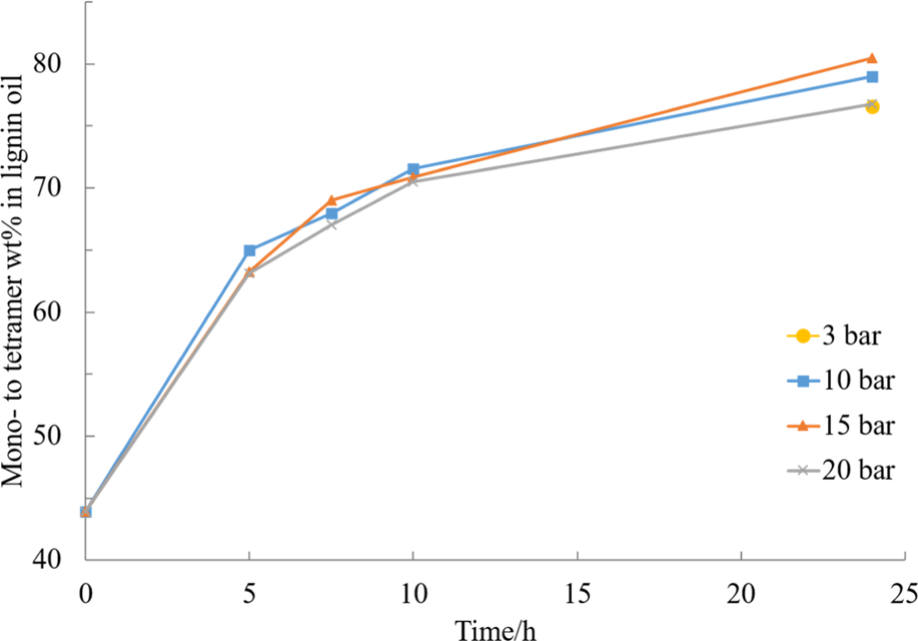
Effect of initial hydrogen pressure on the mono- to tetramer composition of the experiment in EtOH–H_2_O (50/50, v/v) mixture catalyzed by 5% Ru/C at 240 °C.

As observed in the results presented in Figure 7, only a small difference between the mono- to tetramer yield from reactions under different H_2_ pressure during the experiment after 24 h was observed. This indicates that lower concentrations of H_2_ introduced into the reaction mixture were already sufficient to result in similar hydrogenolysis and hydrogenation as at higher pressures. This would indicate that the catalyst surface was already covered with hydrogen at a pressure of 3 bar and no additional benefit was brought to the lignin products by increasing the pressure, as negligible influence on the yield and selectivity was observed. In addition, the results also show the non-competitive adsorption of lignin and hydrogen on the catalyst surface.

**Figure 8:**
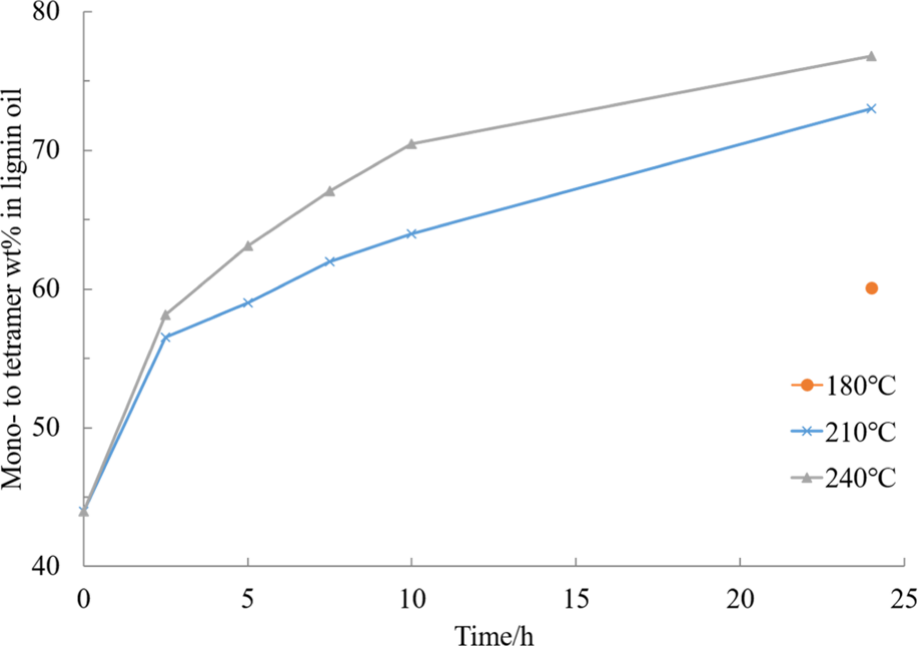
Effect of temperature on the mono- to tetramer composition of the experiment in EtOH–H_2_O (50/50, v/v) mixture catalyzed by 5% Ru/C under 20 bar H_2_.

The samples of three experiments conducted in EtOH–H_2_O (50/50, v/v) at different temperatures (180, 210 and 240 °C) were analyzed by different chromatographic methods. By comparing the HPSEC results of the experiments, it was evident that an elevated temperature, at least 210 °C, was required to ensure the efficient conversion of the substrate, as the mono- to tetramer yield at 180 °C was analyzed to be only 60% (Figure 8). However, a smaller difference was observed between the kinetics obtained at 210 °C and 240 °C, although the reaction rates at 240 °C was obviously higher than that at 210 °C. Somewhat more monomers to tetramers (77% compared to 73%), as well as a slightly reduced polymeric fraction were observed after a 24 h experiment at 240 °C compared to 210 °C. A decrease in the average molecular weight (*M*_w_) of lignin oil products from 834 to 677 g/mol and further to 595 g/mol was observed with HPSEC when going to higher reaction temperature. Moreover, GC-FID/TCD results showed that somewhat more gaseous products were formed at higher temperatures, and the content of CO_2_, CO, C_3_H_8_, and C_4_H_10_ increased. The temperature 240 °C was chosen as a compromise between selectivity and kinetics for the majority of the experiments.

**Figure 9:**
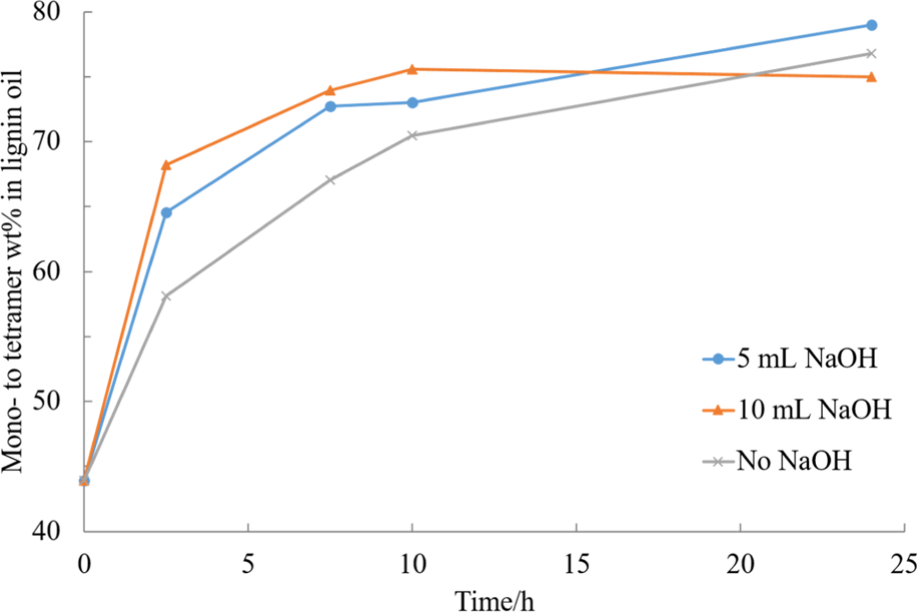
Effect of NaOH on the mono- to tetramer composition of the experiment in EtOH–H_2_O (50/50, v/v) mixture catalyzed by 5% Ru/C under 20 bar H_2_ and 240 °C.

Increasing pH has been proven to have a positive influence on the RCD processes in previous studies,^27,28^ where it has been observed to result in better selectivity for C–O bond cleavage during hydrogenolysis, reduced benzene ring hydrogenation, enhanced depolymerization into aromatic monomers, and decreased amount of residual solid. The samples of two experiments with different amounts of sodium hydroxide (NaOH) added to the reaction medium were compared to a standard RCD conducted under otherwise identical reaction conditions, as shown in Figure 9. The results showed that higher pH accelerated the kinetics at early stages of the experiment. However, the benefit of the increased pH was concluded not to overweigh the problems caused by the salts in subsequent separation.

#### Effect of Catalyst to Substrate Ratio

**Figure 10:**
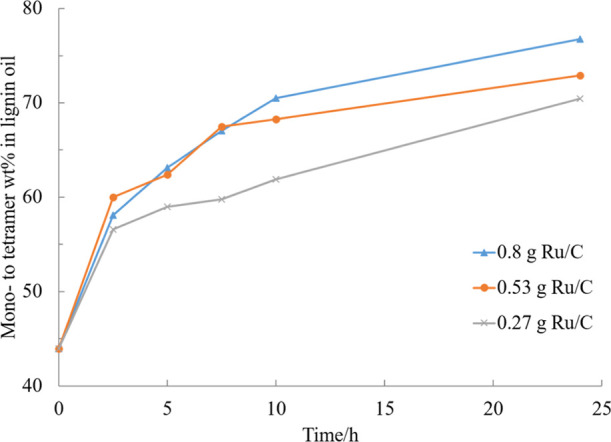
Effect of catalyst loading on the mono- to tetramer composition of the experiment in EtOH–H_2_O (50/50, v/v) mixture catalyzed by 5% Ru/C under 20 bar H_2_ and 240 °C.

The catalytic performance was also studied by decreasing the catalyst loading from the normally used 0.8 g. An equivalent of 0.27 g, 0.53 g, and 0.8 g 5% Ru/C catalyst (dry) were tested, while keeping the lignin amount at 1 g. The reaction conditions were kept identical. The results showed that a higher yield of the mono- to tetramers fraction was obtained with a higher concentration of catalyst. The final mono- to tetramer yield decreased from 77 to 70%, when the catalyst amount was decreased threefold (Figure 10). The difference was especially great when only 0.27 g of the catalyst was used, however, the difference in kinetics was not linearly dependent on the catalyst amount. The reason for this behavior is most likely that the amount of available sites on the catalyst are more than enough compared to the concentration of the lignin macromolecules with 0.8 g of the catalyst. When the reaction progresses and more (moles) oligomers are present in the reaction mixture, a difference is already noticed between 0.8 and 0.53 g, as the amount of available sites starts limiting the observed reaction rate. The greatest difference already from the start of the experiment is noticed analogously with 0.27 g of the catalyst. The gap of catalytic efficiency between 0.53 and 0.8 g started to be visible first after 10 h of reaction.

The loading of lignin and the amount of 5% Ru/C were increased so that the catalyst/substrate ratio was maintained constant. The distribution of monomer and dimers did not differ significantly and the kinetics of the reactions were practically identical. This also confirms that the solubility did not limit the depolymerization rate.

#### Effect of Solvent and Thermal Degradation

**Figure 11:**
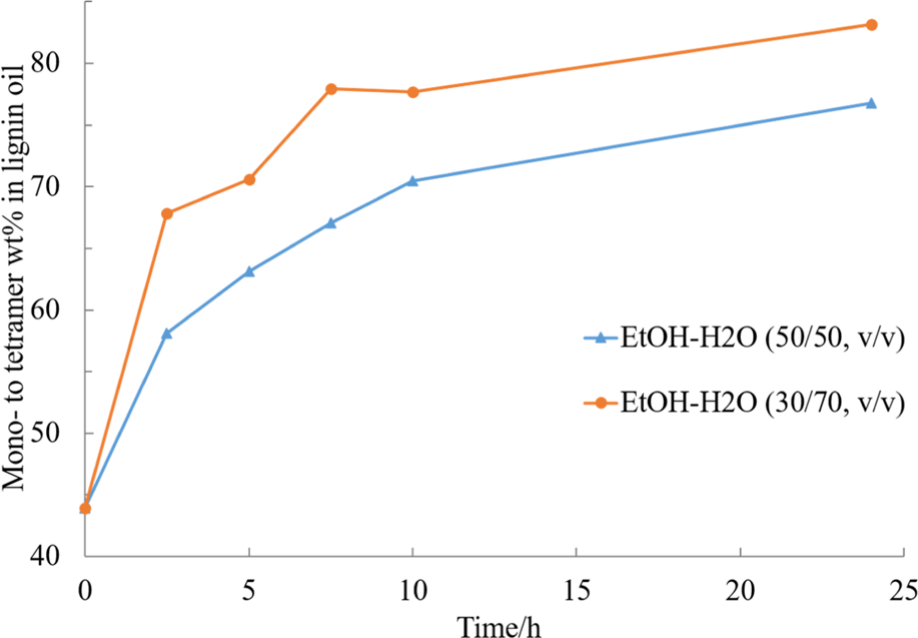
Effect of ethanol–water ratio on the mono- to tetramer composition of the experiment catalyzed by 5% Ru/C under 20 bar H_2_ and 240 °C.

The effect of water in the reaction medium was investigated by comparing the conversion of the substrate in EtOH–H_2_O solution mixed in different volume ratios (30/70, 50/50), see Figure 11. By comparing the results of lignin oil products taken during the experiments, it was observed that an increase in the water content enhanced the depolymerization kinetics. However, the solubility of the lignin in water prevents the use of very high water concentrations. Experiments in EtOH–H_2_O mixtures of volume ratios 30/70 and 50/50 produced final yields of 83 and 77% of mono- to tetramers. The higher polarity of the reaction medium caused by increased water proportion in the mixture seemed to favor the RCD process by enhancing the depolymerization of the lignin oligomers to mono- and dimers.^29,30^

**Figure 12:**
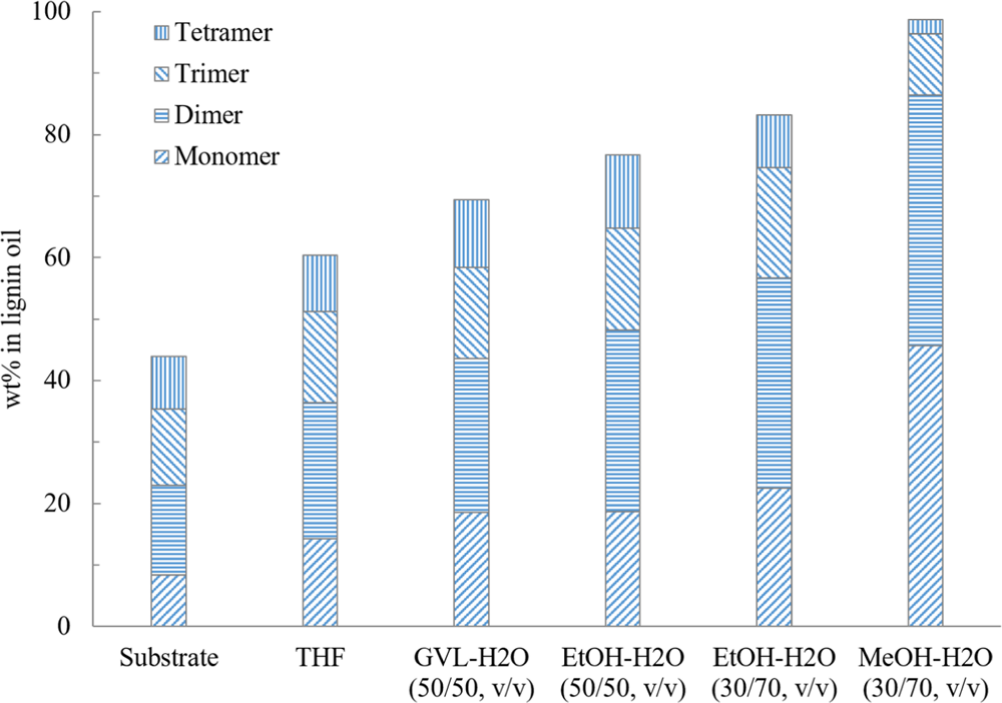
Effect of solvent type on the mono- to tetramer composition after a 24 h experiment catalyzed by 5% Ru/C under 20 bar H_2_ and 240 °C.

The influence of different solvents, including EtOH–H_2_O, MeOH–H_2_O, GVL–H_2_O, THF, and H_2_O, on the conversion of lignin in the RCD system were also studied. The order of decreasing Mw can be observed to be THF → GVL-H_2_O (50/50, v/v) → EtOH–H_2_O (all ratios) → MeOH–H_2_O (30/70, v/v), which is consistent with the order of increase in the yield of desired products (Figure 12). Experiments in THF produced 60% of mono- to tetramers, while experiment in MeOH–H_2_O (30/70, v/v) resulted in the highest yield (98%). The good performance of MeOH–H_2_O mixture was attributed to its higher polarity and the ability of MeOH to dissolve lignin caused by its smaller molecular volume and greater hydrogen bonding ability.^31^ MeOH most probably also acted as a hydrogen donor,^32^ which is advantageous to the catalytic hydrogenolysis and hydrogenation processes. The reaction in THF displayed the lowest depolymerization toward smaller aromatic compounds, as the unstable monomers and small oligomers were more likely to undergo repolymerization in tetrahydrofuran.

**Figure 13:**
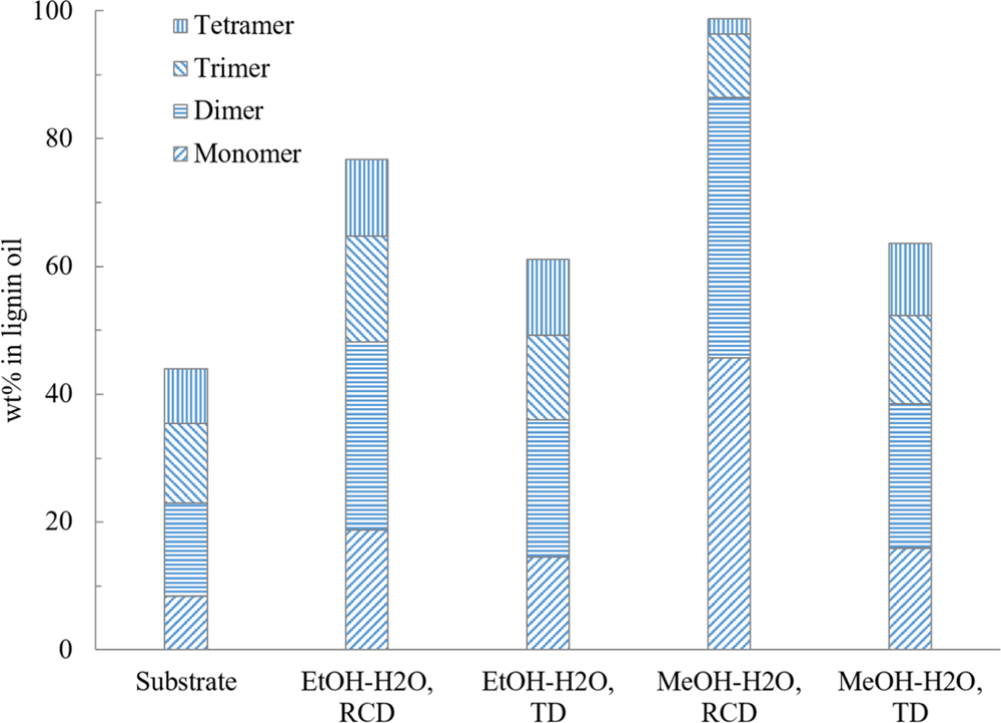
Comparison of the mono- to tetramer composition after 24 h thermal degradation (TD) and standard RCD experiments at 240 °C. RCD reaction employed 5% Ru/C and 20 bar H_2_ while TD utilized 10 bar Ar without a catalyst.

Two non-catalytic reactions were carried out in MeOH–H_2_O (30/70, v/v) and EtOH–H_2_O (50/50, v/v) under an argon atmosphere at 240 °C to study the TD (Figure 13). It is observed that when the catalyst and hydrogen are used, both conversion and yield, especially the yields of monomers and dimers, which are the main targets of this work, are substantially higher. This indicates that a reductive atmosphere and catalyst significantly promoted the depolymerization. For example, almost 100% of mono- to tetramers were obtained from the RCD experiment performed in methanol–water mixture, whereas a thermally treated liquid product contains about 63% mono- to tetramers. It can also be concluded that lignin oil from the RCD process at 240 °C is mainly a result of hydrogenolysis and hydrogenation catalyzed by a redox catalyst, but that TD also plays a role in the depolymerization partly depending on the solvent. ^13^C NMR analysis also confirmed this by individual sharper peaks of products compared with the raw material, indicating that the lignin fragments are cleaved into smaller molecules (Figure S6).

#### Lignin Oil Mass Balance

The mass balance defined in eq 1 was studied by performing experiments in EtOH–H_2_O (50/50, v/v) under 20 bar H_2_ and 240 °C without taking intermediate samples. The results shown in Table 1 indicated around 70% liquid product yield for standard RCD experiments and above 90% for non-catalytic experiments. The difference in the yield might be caused by (1) char formation by random repolymerization of the active radicals^33,34^ and (2) production of gaseous products by either decarbonylation/decarboxylation or cleavage of aliphatic side chains and ring substituents during catalytic hydroprocessing.^35^

**Table 1:** Mass Balance of Catalytic and Non-catalytic Experiments

exp.	substrate/g	lignin oil/g	mass balance/%	average mass balance/%
Catalytic^a^				
1	0.9998	0.7022	70.24	71.62
2	1.0005	0.7472	74.68	
3	0.9996	0.6990	69.93	
Non-catalytic^b^				
1	1.0005	0.9093	90.88	91.96
2	1.0001	0.9147	91.46	
3	1.0000	0.9374	93.74	
4	1.0001	0.9091	90.90	
5	1.0003	0.9486	94.83	
6	1.0005	0.9000	89.96	

a Reaction conditions: EtOH–H_2_O (50/50, v/v), Ru/C, 20 bar H_2_, 240 °C.

b Reaction conditions: EtOH–H_2_O (50/50, v/v), 20 bar H_2_, 240 °C.

The gaseous products of RCD reactions were analyzed by GC-FID/TCD. The peaks were identified by GC–MS. The main gaseous product from RCD processes was methane produced by hydrogenolysis of the Ph–OCH_3_ group, which accounts for around 85% of the total gas. Other gaseous produced include ethane, propane, butane, and CO_2_, which were also observed in other works.^18,35^ The composition of minority gases varies according to the reaction conditions, especially to the solvent utilized. For example, the reaction in THF was observed to have produced more ethane, while more butane was formed with the GVL–H_2_O mixture. After the TD reactions in MeOH–H_2_O (30/70, v/v) and H_2_O, in addition to the gases mentioned above, some CO was also detected. Thermal degradation in MeOH–H_2_O also produced a little more ethane and propane.

### Structural Characterization of Raw Material and Reaction Products by NMR

A qualitative analysis by 2D HSQC NMR, ^13^C NMR and quantitative analysis by ^31^P was performed on samples from the TD and RCD reactions. These were compared to the starting material, and the results are shown in Figures S6–S8. The studied samples from the RCD reaction used the following process parameters: Ru/C with 20 bar H_2_ and the samples from TD reaction used no catalyst with 10 bar Ar. Both the RCD and TD reactions were conducted in EtOH–H_2_O (50/50, v/v) and MeOH–H_2_O (30/70, v/v) solvent mixtures. From the HSQC-spectrum of the starting material (Figure S7e), the *C*–H correlation peaks from both syringyl and guaiacyl units, enol ether, and fatty acids could be detected in the aromatic region, approximately δ_C_/δ_H_ (100–140/5.00–8.00) ppm. In the oxygenated aliphatic region, δ_C_/δ_H_ (50–90/2.50–5.00) ppm, the correlation peaks corresponding to signals from the −OMe groups, β–β substructure, carbohydrate impurities, and the aryl glycerol end group were detected. In the aliphatic region δ_C_/δ_H_ (50–0/3.00–0) ppm, the main correlation peaks were from fatty acid impurities. In all four of the processed samples, the identifiable correlation peaks, besides the −OMe group, in the oxygenated aliphatic region were removed. Considerable amounts of correlation peaks of CH_2_ can be seen below the −OMe correlation peak. These most likely originate from primary alcohols that can have been formed from partially dehydroxylated side chains. The aliphatic region consists of four clusters of correlation peaks (Figure S7a), of which two consists of CH/CH_3_ at approximately δ_C_/δ_H_ (25.0–7.0/1.50–0.60) and δ_C_/δ_H_ (23.0–7.0/2.65–1.70) ppm and two consists of CH_2_ at δ_C_/δ_H_ (38.5–22.5/2.05–1.00) and δ_C_/δ_H_ (42.5–26.5/3.05–2.05) ppm. The chemical shifts of these clusters are in agreement with previously published shifts of saturated aliphatic side chains (methyl, ethyl, and propyl) and also partially dehydroxylated side chains.^36−39^ In the aromatic region, the two samples from the RCD reactions had a slightly lower proton chemical shift compared to the starting material and the thermally treated samples. Correlation peaks at a higher proton shift in the aromatic region are often assigned as aryl rings with oxidized α-positions. The thermally treated samples have a new cluster at δ_C_/δ_H_ (131.5–121.6/7.90–6.80), these signals could originate from stilbene structures, α,β-unsaturated carbonyl structures, demethoxylated lignin units, and some signals from oxidized G-units. As for the samples from RCD reactions, the significant reduction in these structures is most likely due to the reductive process. The incorporation of the solvent is evident from the methyl ester correlation peak at δ_C_/δ_H_ (52.0/3.45) ppm in the samples that used MeOH–H_2_O solvent mixture and the correlation peak of the CH_2_ at δ_C_/δ_H_ (60.5/4.06) ppm in the samples which corresponds to EtOH–H_2_O solvent mixture. The fragmentation of all these processed samples can be seen from the ^13^C NMR due to the considerably sharper peaks compared to the starting material. The signal at approximately 152 ppm is often assigned to C-3/C-5 in etherified S units and has almost completely been removed in the processed samples. The amounts of free hydroxyl groups calculated based on ^31^P NMR results (Figure S8) are listed in Table 2.

**Table 2:** Amount of Free Hydroxyl Groups in mmol/g Based on ^31^P NMR Analysis

	aliphatic	phenolic	S-units^a^	G-units^b^	OH total	COOH
substrate	1.32	3.08	2.31	0.77	4.40	0.66
MeOH–H_2_O, RCD	1.83	4.52	2.92	1.60	6.35	0.48
EtOH–H_2_O, RCD	1.00	3.91	2.77	1.14	4.91	0.41
MeOH–H_2_O, TD	0.87	4.63	3.11	1.52	5.50	0.36
EtOH–H_2_O, TD	0.90	4.11	2.87	1.24	5.01	0.34

a S-units and/or condensed G-units.

b G-units and H-units.

It is evident from the values that the polymeric/lignin molecules are cleaved into smaller compounds, when comparing to the starting material, by producing more free hydroxyl groups during the reaction. Moreover, the amount of carboxyl group was found to be lower after the experiments, which can be explained by the decarboxylation to form gaseous products. The catalytic experiment performed in MeOH–H_2_O system had significantly increased the amount of free aliphatic hydroxyl groups compared to the other experiments.

### Identification of Lignin Oil Products by GC–MS

**Figure 14:**
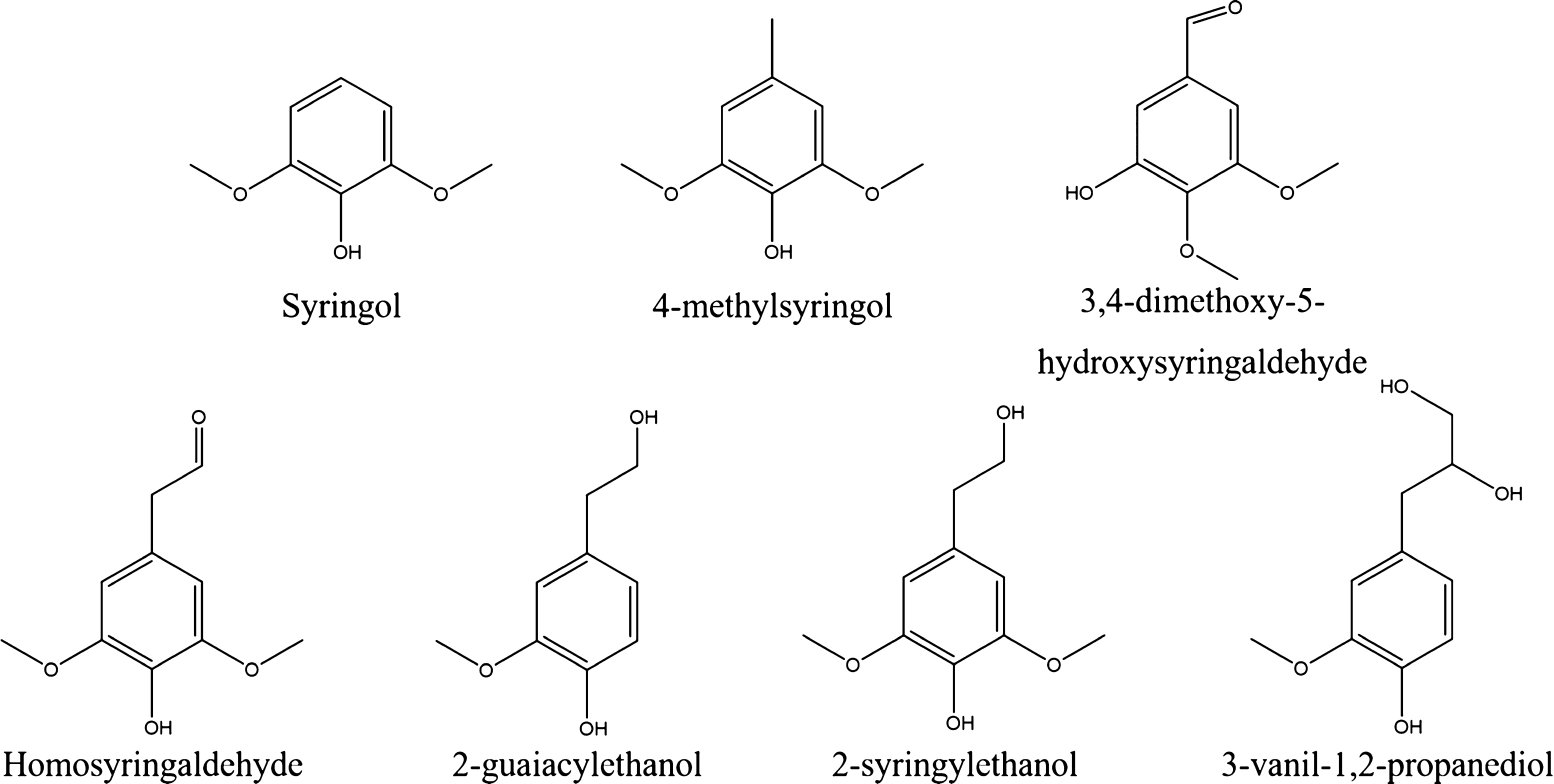
Most abundant monomer structures in the product mixture.

The lignin oil products after a 24 h RCD experiment in EtOH–H_2_O (50/50, v/v) mixture catalyzed by 5% Ru/C under 20 bar H_2_ and 240 °C were also characterized by GC–MS, which is in agreement with NMR results. The monomers identified are shown in the chromatogram in Figure S9. It can be seen from the results that the most abundant monomers among all the compounds detected were syringol, 4-methylsyringol, 3,4-dimethoxy-5-hydroxysyringaldehyde, homosyringaldehyde, 2-guaiacylethanol, 2-syringylethanol, and 3-vanil-1,2-propanediol, which are presented in Figure 14. The results were also confirmed by GC-FID analysis. The structure of the most abundant monomeric products shows that the phenolic OH and methoxy groups remained largely intact during the depolymerization, however, the aliphatic region underwent cleavage of varying extent all the way to being completely removed in the case of syringol.

**Figure 15:**
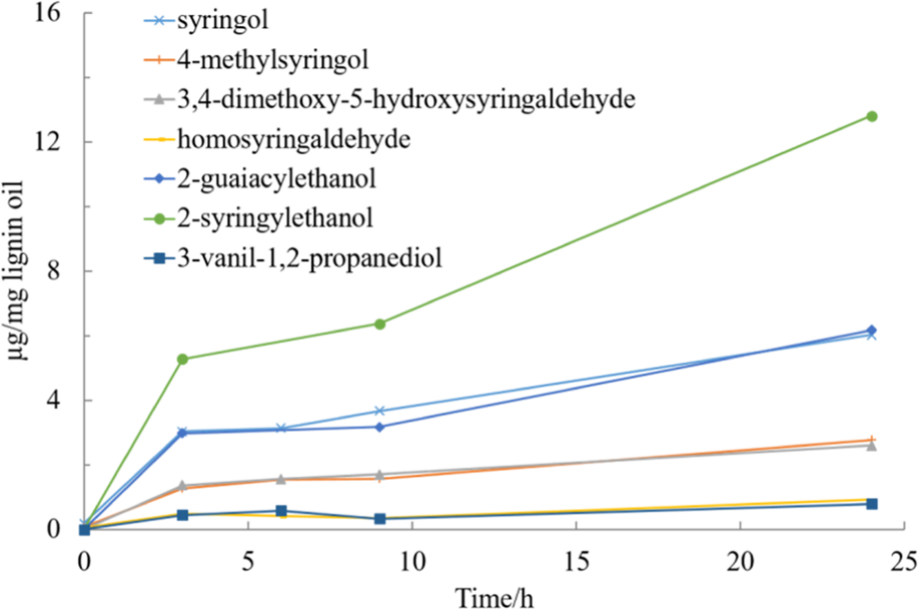
Concentration of the main monomer products as a function of time fora non-catalytic experiment in EtOH–H_2_O (50/50, v/v) mixture under 20 bar H_2_ and 240 °C.

**Figure 16:**
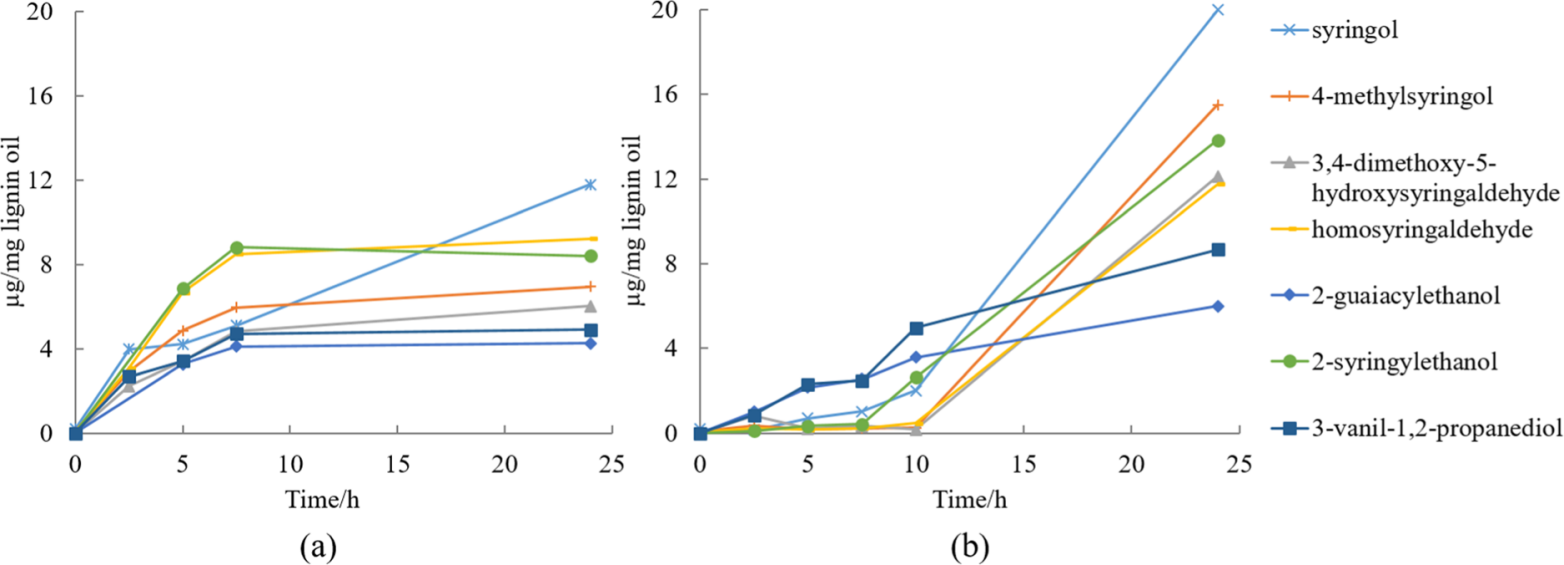
Concentration of the most abundant monomers as a function of time in (a) EtOH–H_2_O (50/50, v/v) and (b) MeOH–H_2_O (30/70, v/v) mixtures catalyzed by 5% Ru/C under 20 bar H_2_ and 240 °C characterized by GC.

The concentrations of the most abundant monomers in experiments performed in ethanol without catalyst are presented in Figure 15 and catalytic experiments in ethanol and methanol mixtures are presented in Figure 16. The results clearly show that the product distribution is different. 2-Syringylethanol, 2-guaiacylethanol, and syringol were the most abundant in the non-catalytic experiment, with 2-syringylethanol being clearly the most common compound present in the mixture. In the catalytic experiments, syringol was the most abundant final product and also other compound, where the aliphatic chain had been more severely cleaved were present in higher concentrations. Moreover, the consecutive reaction pathway also observed previously with HPSEC (Figure 6) was evident especially when the reaction was performed in methanol water mixture, as the concentration of the monomers increased significantly first after 10 h of experiment.

### Characterization of Different Fractions from Ultrafiltration

The lignin oil product after a 24 h experiment in EtOH–H_2_O (50/50, v/v) mixture under 20 bar H_2_ and 240 °C was introduced to an ultrafiltration system equipped with a regenerated cellulose membrane of 1 kDa. The filtrate obtained was dried and analyzed with GC-FID, HPSEC, and different NMR methods.

Structurally, the filtrate was similar to the lignin oil product before ultrafiltration based on 2D HSQC and ^13^C NMR as shown in Figures S10 and S11. However, based on ^31^P NMR, the filtrate contained higher amounts of hydroxyl groups, which was indicated by sharper peaks in the spectra (Figure S12). The concentrations of hydroxyl groups in samples before and after ultrafiltration were determined to be 4.91 and 6.44 mmol/g, respectively. Moreover, the molecular weight was determined to be 434 g/mol, corresponding to DP of about 3 with HPSEC (Figure S13). Quantitative GC results showed that the concentration of identified monomers and dimers was nearly twice that of the lignin oil product’s before ultrafiltration.

The large molecule fractions in the residue before and after a further oxidation experiment were analyzed by HPSEC, method B, as shown in Figure S13. The results indicated that the large molecule fractions obtained from the RCD process cannot be further degraded efficiently, at least by oxidation. The average molecular weight was only slightly decreased from 1590 to 1292 g/mol.

## Conclusions

Reductive catalytic depolymerization was performed on lignin from a novel semi-industrial biorefinery process using Ru/C, Pd/C, and Ni/Al_2_O_3_ catalysts. The lignin macromolecules were depolymerized to mono- and dimers with a continuous decline in average molecular mass over reaction time, showing consecutive reaction pathways. The redox catalyst and hydrogen gas were essential for achieving selective depolymerization and high product stability and yield. The reaction kinetics were strongly promoted by elevated temperatures; however, no significant difference was observed with increased pressure. The composition of the reaction medium significantly affected the reaction products with aqueous mixtures of ethanol and methanol providing the highest yields. The highest obtained yield of the mono- to tetramers fraction was 98% in the liquid phase products and the yield of the mono and dimers fraction was over 85%. Gaseous products, mainly CO_2_, CH_4_, and short alkanes were also formed under the studied conditions. The developed method was shown to be efficient for obtaining a monomer—short oligomer fraction from an industrial lignin. The substrate was obtained utilizing a novel fractionation process that employs mild conditions to extract pure fractions of hemicellulose, cellulose, and lignin. The lignin is very pure, relatively soluble, and sulfur-free, which is very beneficial for following catalytic valorization steps. The current work contributes significantly to the development of a sustainable biorefinery process by enabling the production of lignin monomers and short oligomers for further valorization. The overall process including the fractionation of the biomass followed by further processing is very well aligned with the principles of green engineering and chemistry, as almost 100% of the renewable feedstock is utilized for products and recirculation as well as energy efficiency of the aqueous based process are on high level.

## Supporting Information Available

Characterization of lignin oil products after a 24 h RCD experiment in EtOH–H_2_O (50/50, v/v) mixture by LC/MS (S1), retention time of different molecular weight compounds in HPSEC (S2), concentration of lignin in EtOH–H_2_O (50/50, v/v) under 20 bar H_2_ without the catalyst (S3), effect of sampling on the mono- to tetramer composition after a 24 h experiment (S4), effect of dissolution kinetics on the mono- to tetramer composition of experiment (S5), characterization of raw material and lignin oil products by ^13^C NMR (S6), characterization of raw material and lignin oil products after 24 h TD and standard RCD experiments in MeOH–H_2_O (30/70, v/v) and EtOH–H_2_O (50/50, v/v) by 2D HSQC NMR (S7), characterization of raw material and lignin oil products after 24 h TD and standard RCD experiments in MeOH–H_2_O (30/70, v/v) and EtOH–H_2_O (50/50, v/v) by ^31^P NMR (S8), identification of lignin monomers in lignin oil products after a 24 h RCD experiment in EtOH–H_2_O (50/50, v/v) mixture by GC/MS (S9), characterization of the lignin oil products before and after ultrafiltration by 2D HSQC NMR (S10), characterization of the lignin oil products before and after ultrafiltration by ^13^C NMR (S11), characterization of the lignin oil products before and after ultrafiltration by ^31^P NMR (S12), and characterization of the lignin oil products before and after ultrafiltration and the products after oxidation of residue by HPSEC (S13) (PDF)
