# Supplementary material for: Reductive Catalytic Depolymerization of Semi-industrial Wood-Based Lignin
Source: Ind Eng Chem Res. 2021 Nov 9;60(47):16827–38. doi: 10.1021/acs.iecr.1c03154 (PMC8641393; doi:10.1021/acs.iecr.1c03154)
Supplement: Supplementary file 1 — ie1c03154_si_001.pdf [file ie1c03154_si_001.pdf]

Reductive catalytic depolymerization of semi-industrial wood-based lignin

*Xiaojia Lu[1,2], Lucas Lagerquist[3], Kari Eränen[1], Jarl Hemming[4], Patrik Eklund[3], Lionel Estel[2], Sebastien Leveneur[1,2], Henrik Grenman[1]**

[1]Laboratory of Industrial Chemistry and Reaction Engineering, Johan Gadolin Process Chemistry Centre, Åbo Akademi University, Biskopsgatan 8, 20500 Turku, Finland;

[2]Normandie Univ, INSA Rouen, UNIROUEN, LSPC, EA4704, 76000 Rouen, France;

[3]Laboratory of Molecular Science and Engineering, Johan Gadolin Process Chemistry Centre, Åbo Akademi University, Biskopsgatan 8, 20500 Turku, Finland;

[4]Laboratory of Wood and Paper Chemistry, Johan Gadolin Process Chemistry Centre, Åbo Akademi University, Porthansgatan 3, 20500 Turku, Finland.

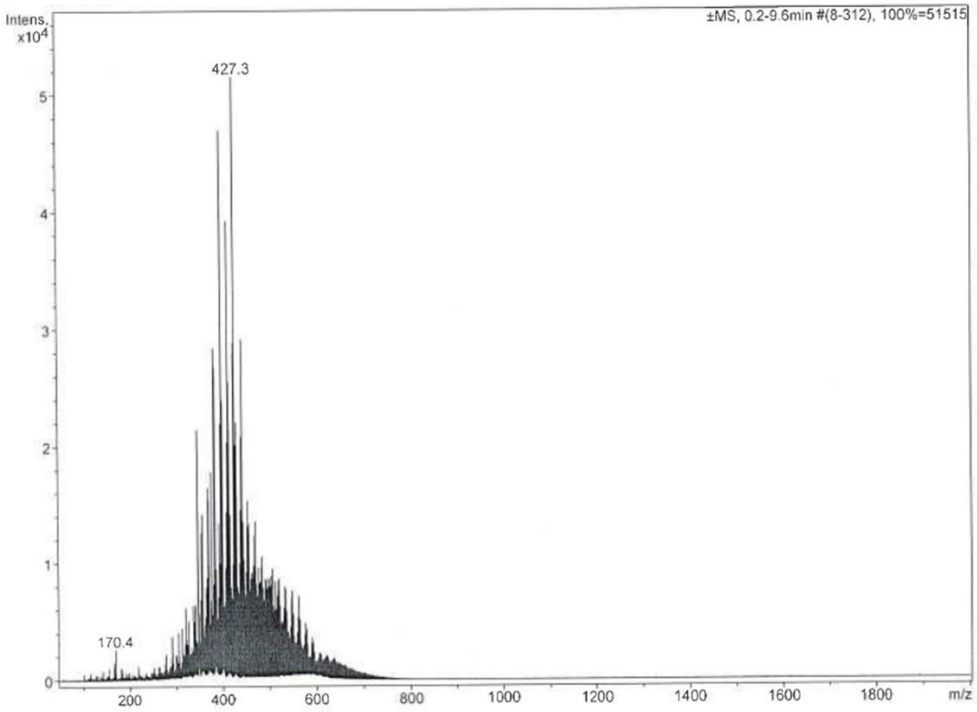

Figure S1. Characterization of lignin oil products after a 24 hours RCD experiment in EtOH-H_2O (50/50, v/v) mixture catalyzed by 5% Ru/C under 20 bar H_2 and 240 °C by LC/MS.

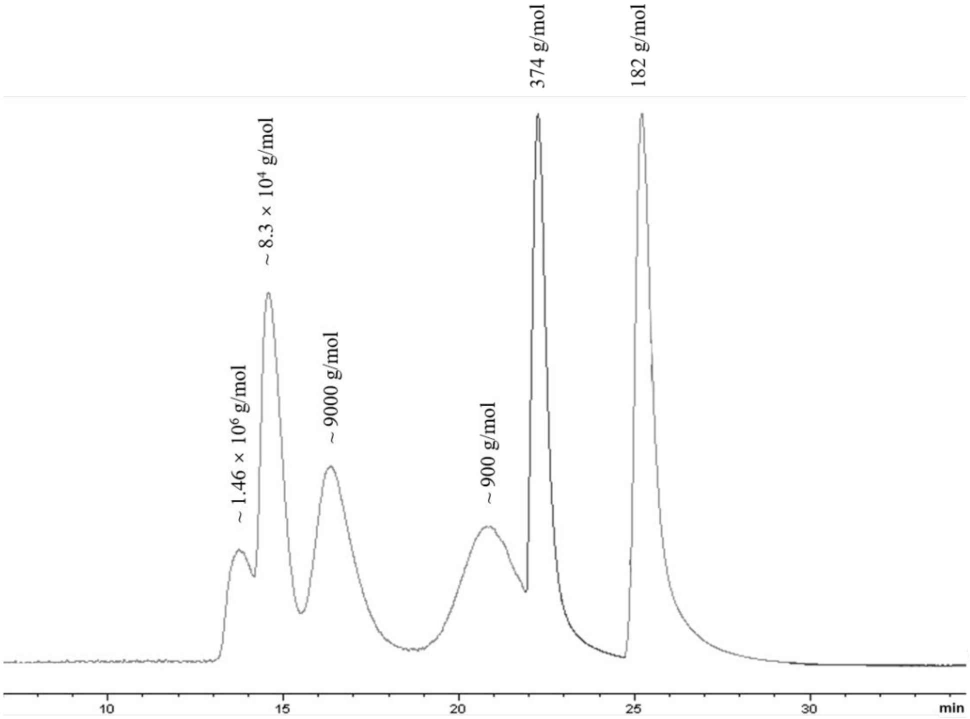

Figure S2. Retention time of different molecular weight compounds in HPSEC, method A.

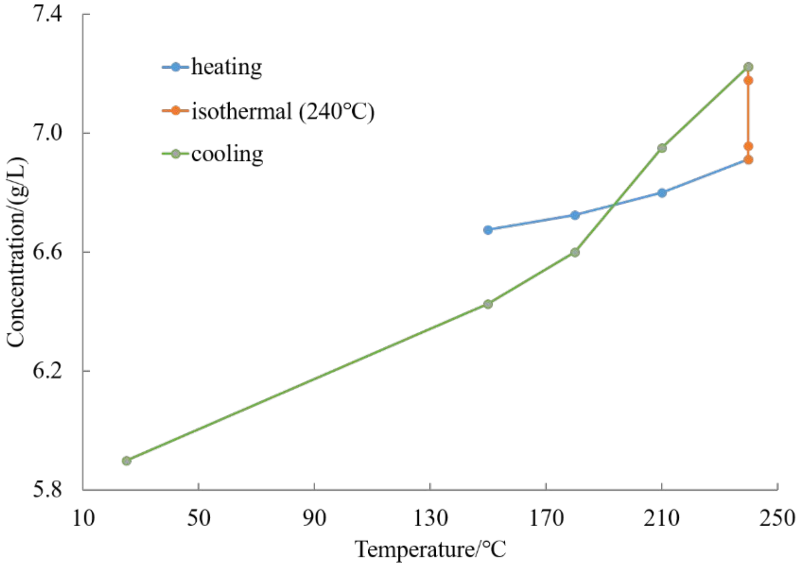

Figure S3. Evolution of concentration of lignin in EtOH-H_2O (50/50, v/v) under 20 bar H_2 without utilizing catalyst.

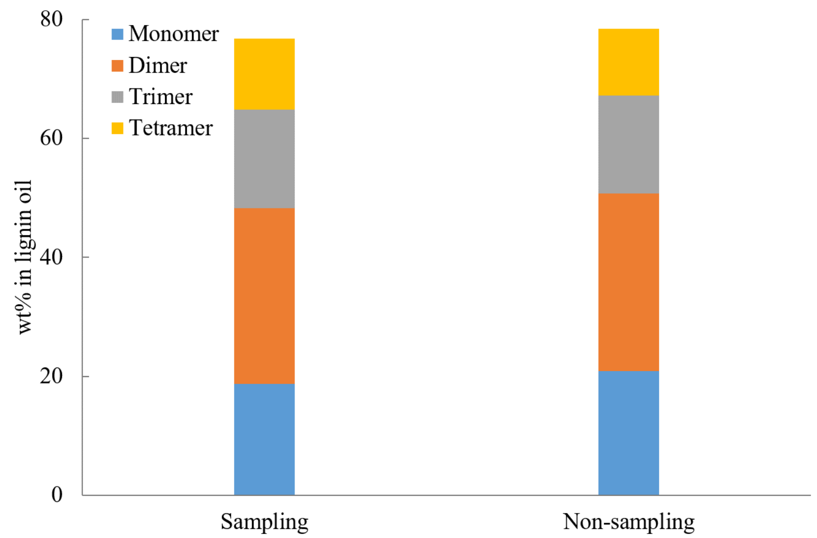

Figure S4. Effect of sampling on the mono- to tetramer composition after a 24 hours experiment in EtOH-H_2O (50/50, v/v) mixture under 20 bar H_2 and 240 °C.

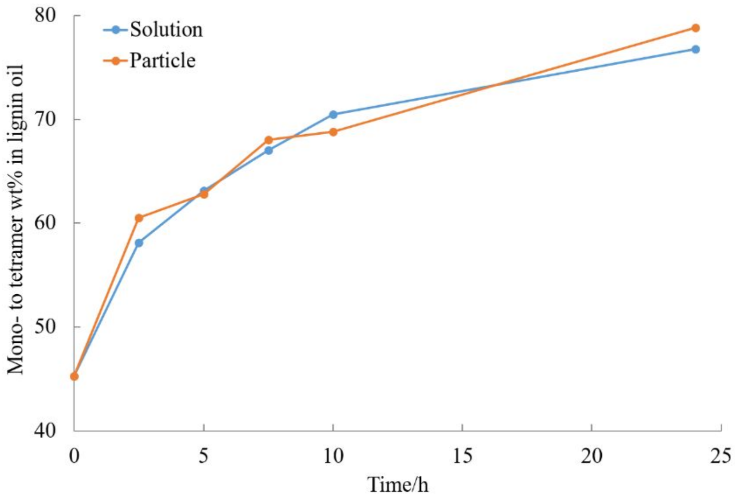

Figure S5. Effect of dissolution kinetics on the mono- to tetramer composition of experiment in EtOH-H_2O (50/50, v/v) mixture under 20 bar H_2 and 240 °C.

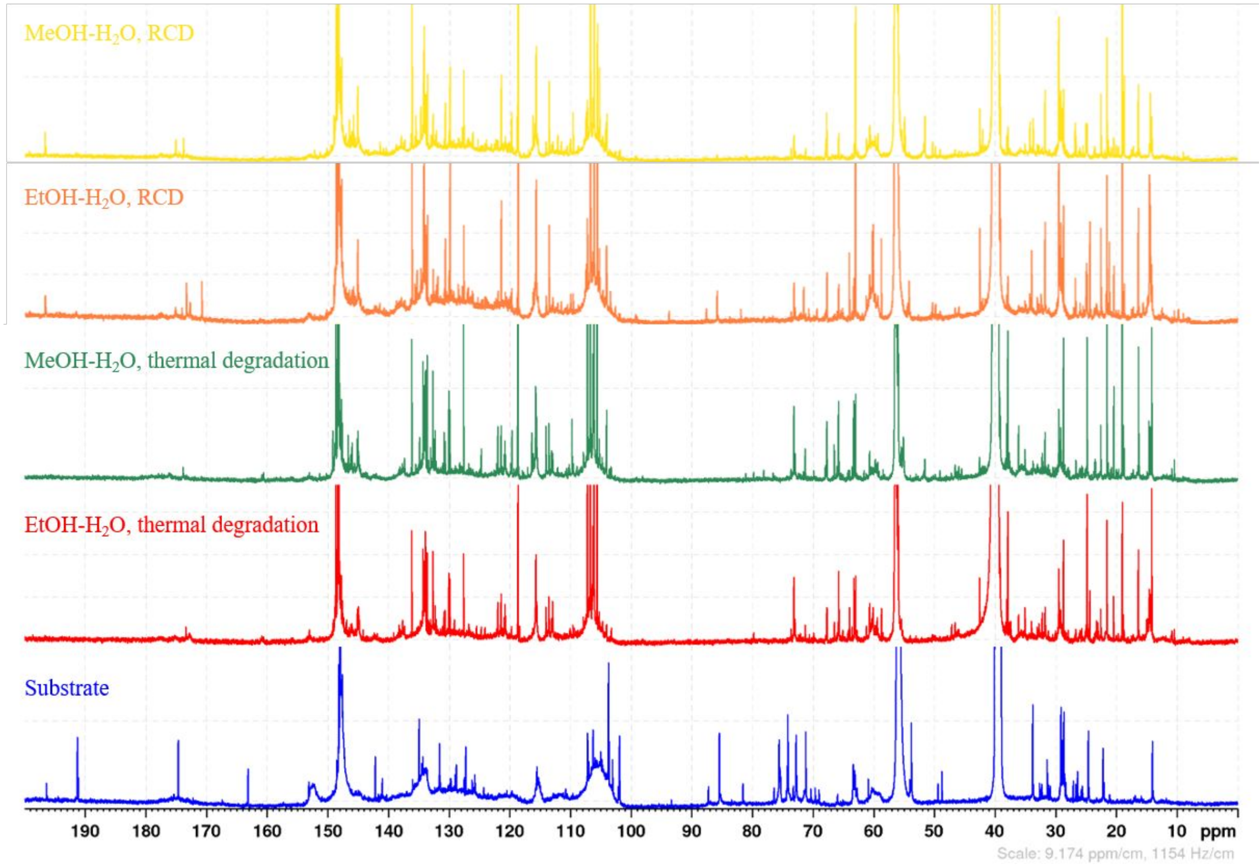

Figure S6. Characterization of raw material and lignin oil products after 24 hours thermal degradation and standard RCD experiments in MeOH-H_2O (30/70, v/v) and EtOH-H_2O (50/50, v/v) at 240 °C by ^{13}C NMR.

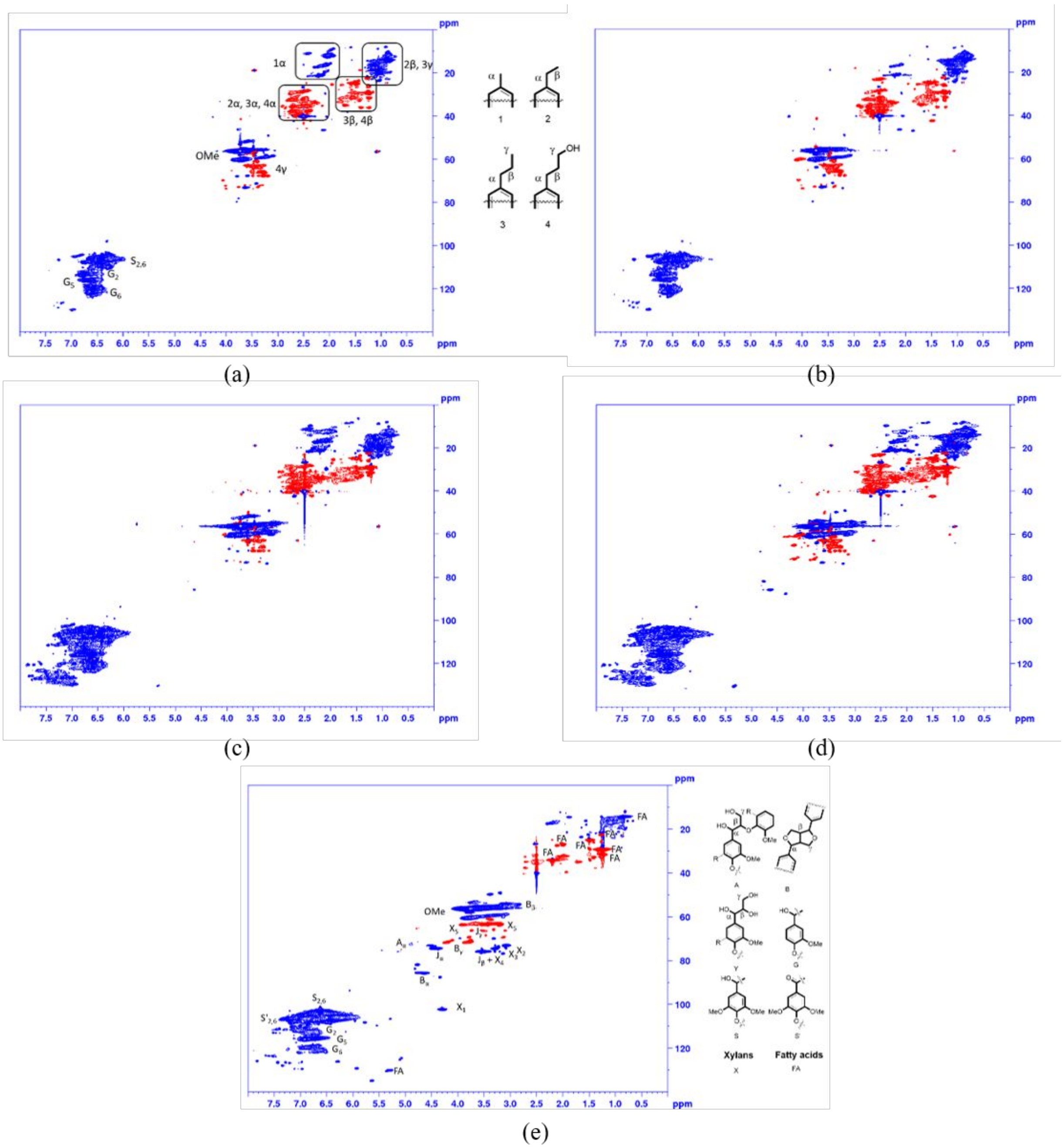

Figure S7. Characterization of raw material and lignin oil products after 24 hours thermal degradation and standard RCD experiments in MeOH-H_2O (30/70, v/v) and EtOH-H_2O (50/50, v/v) at 240 °C by 2D HSQC NMR. (a) MeOH-H_2O, RCD; (b) EtOH-H_2O, RCD; (c) MeOH-H_2O, thermal degradation; (d) EtOH-H_2O, thermal degradation; (e) substrate.

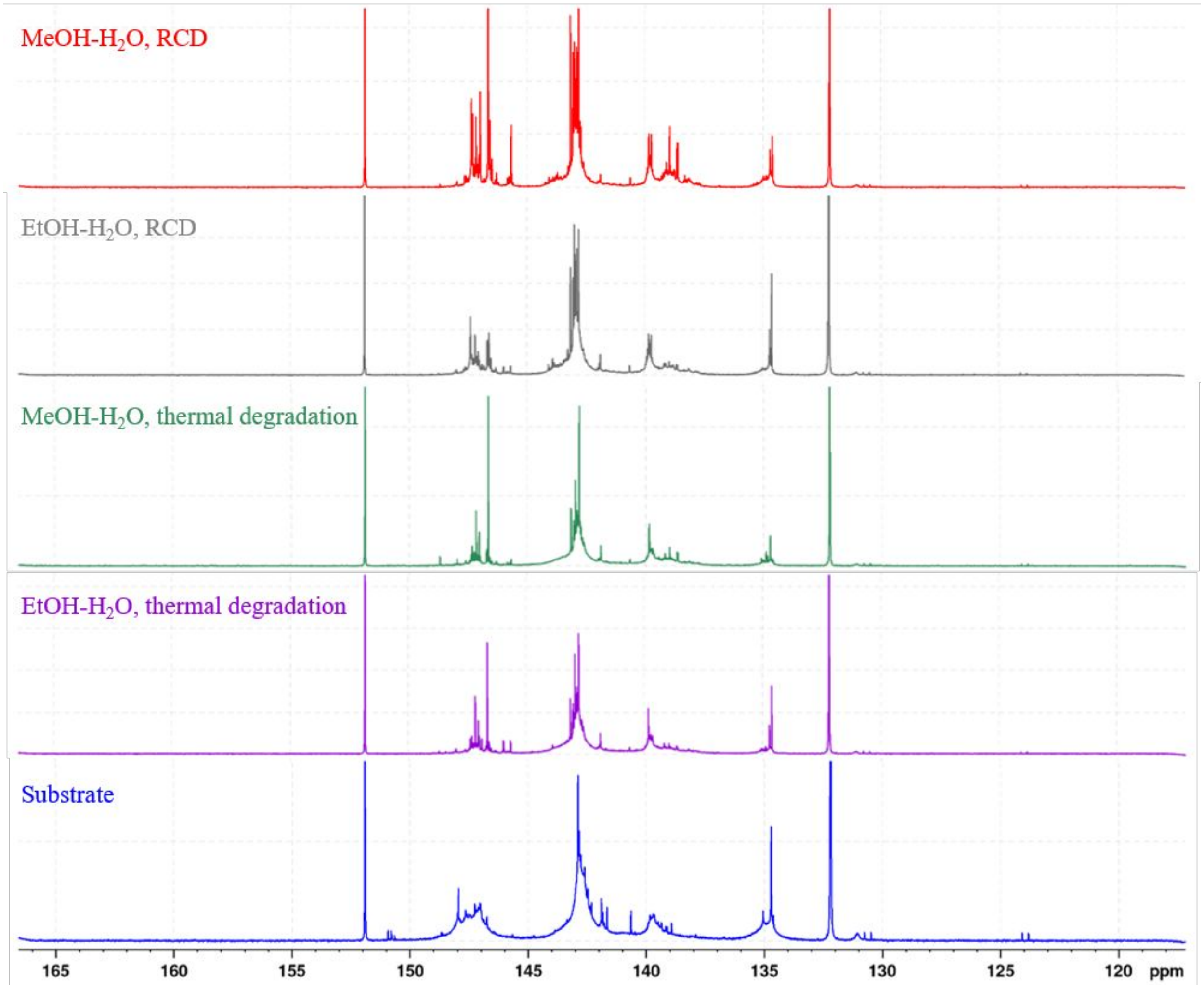

Figure S8. Characterization of raw material and lignin oil products after 24 hours thermal degradation and standard RCD experiments in MeOH-H_2O (30/70, v/v) and EtOH-H_2O (50/50, v/v) at 240 °C by ^{31}P NMR.

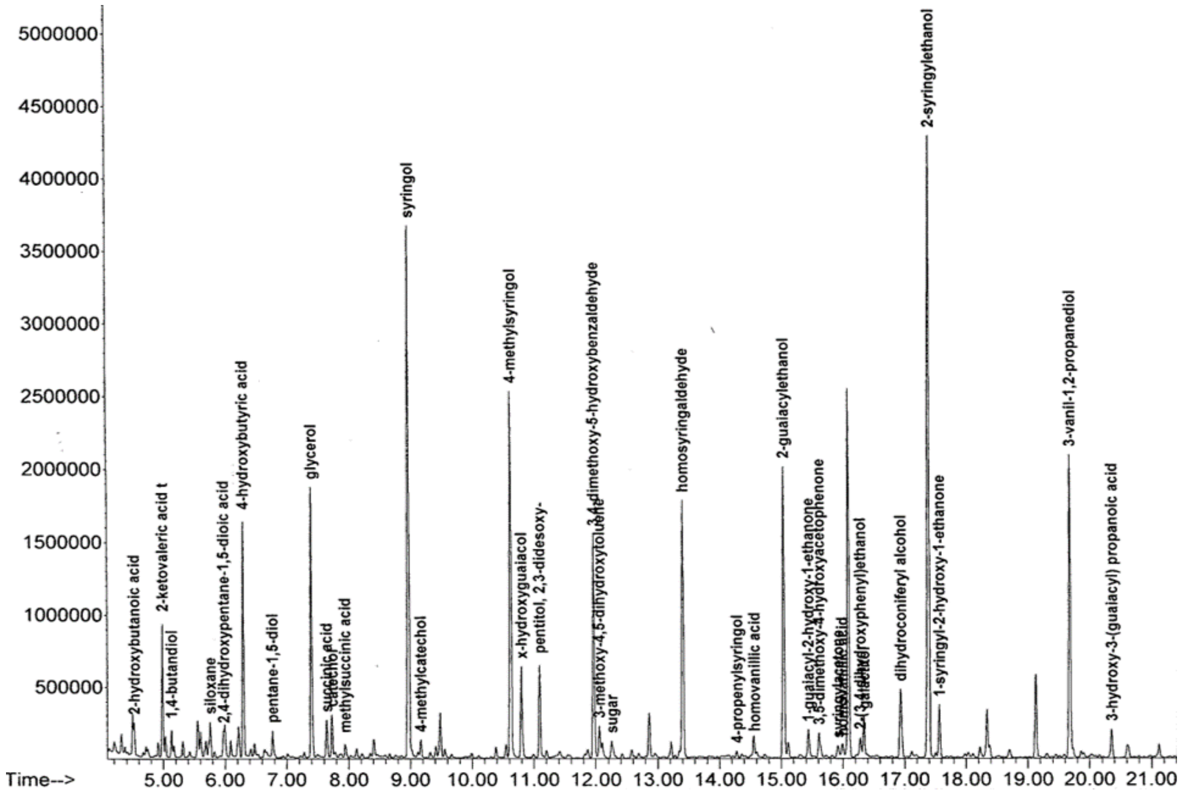

Figure S9. Identification of lignin monomers in lignin oil products after a 24 hours RCD experiment in EtOH-H_2O (50/50, v/v) mixture catalyzed by 5% Ru/C under 20 bar H_2 and 240 ° C by GC/MS.

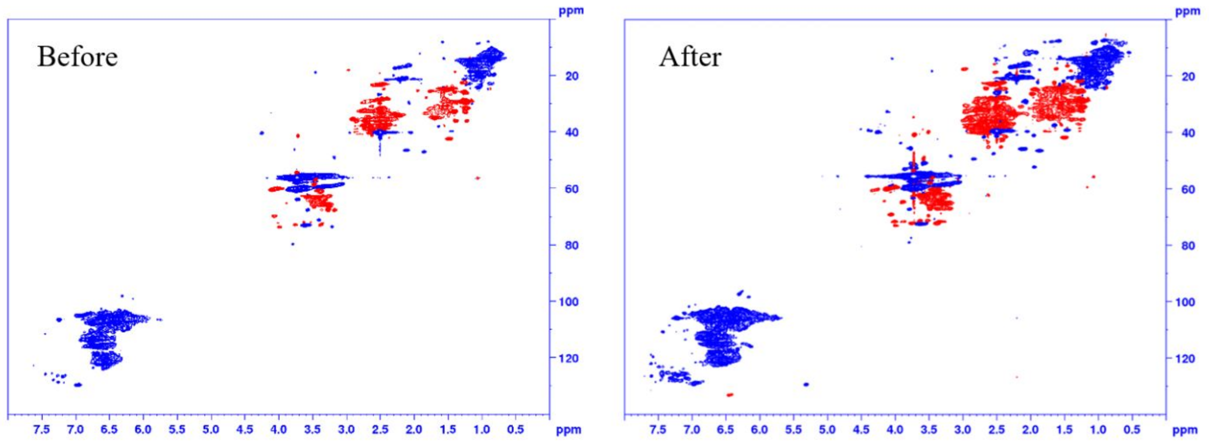

Figure S10. Characterization of the lignin oil products before and after ultrafiltration by 2D HSQC NMR. The samples were from a 24 hours experiment in EtOH-H_2O (50/50, v/v) mixture catalyzed by 5% Ru/C under 20 bar H_2 and 240 °C.

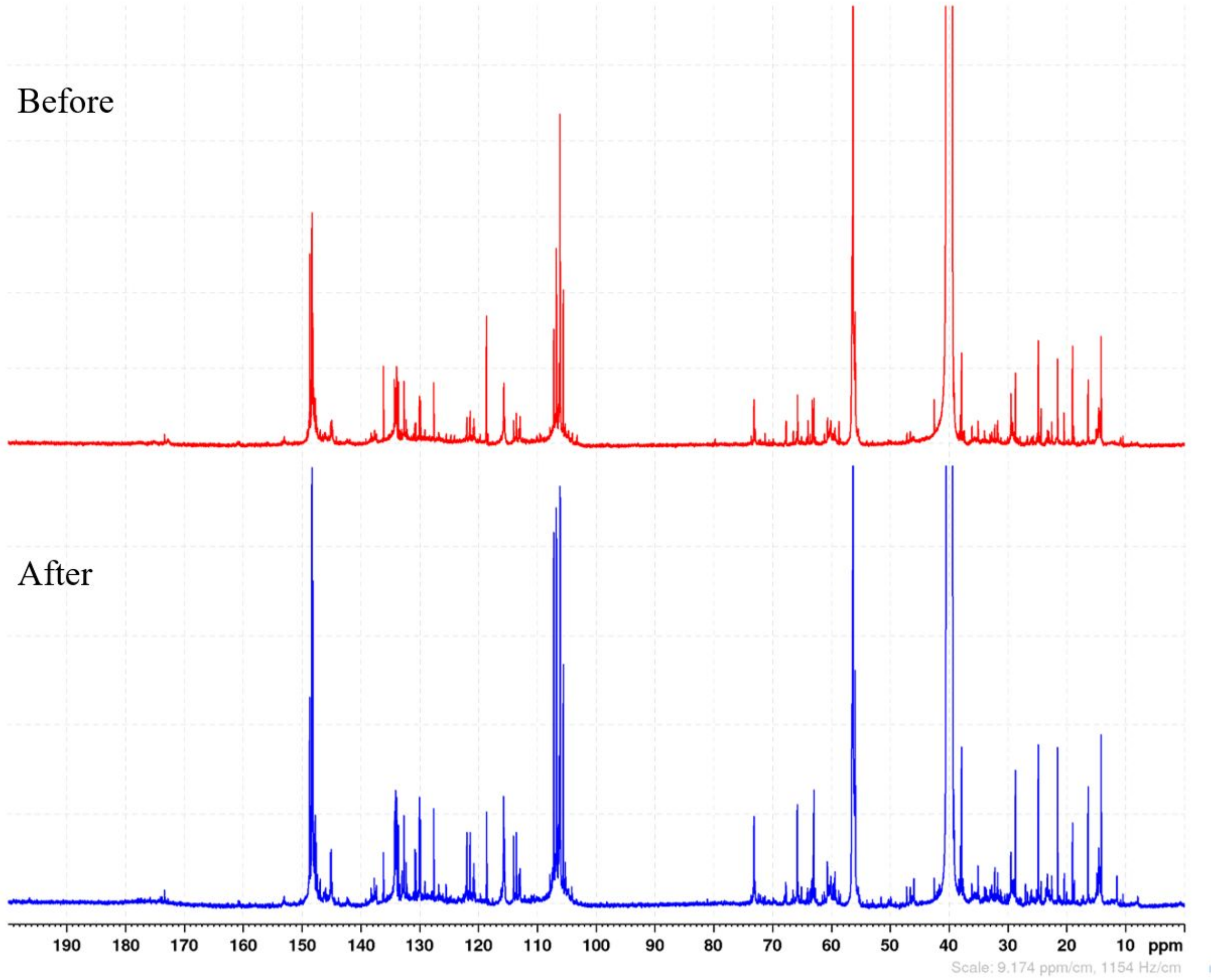

Figure S11. Characterization of the lignin oil products before and after ultrafiltration by ^{13}C NMR. The samples were from a 24 hours experiment in EtOH-H_2O (50/50, v/v) mixture catalyzed by 5% Ru/C under 20 bar H_2 and 240 °C.

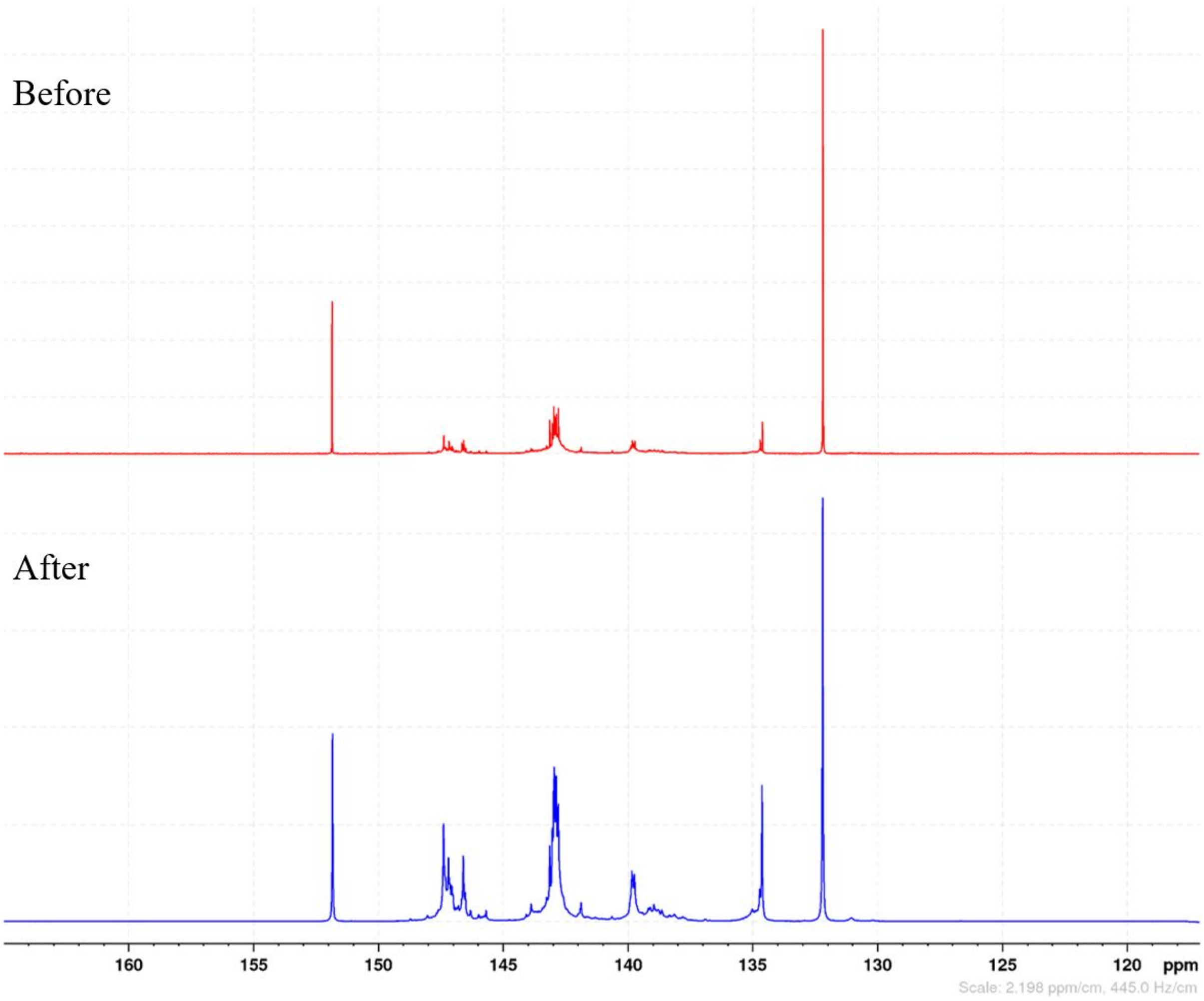

Figure S12. Characterization of the lignin oil products before and after ultrafiltration by ^{31}P NMR. The samples were from a 24 hours experiment in EtOH-H_2O (50/50, v/v) mixture catalyzed by 5% Ru/C under 20 bar H_2 and 240 °C.

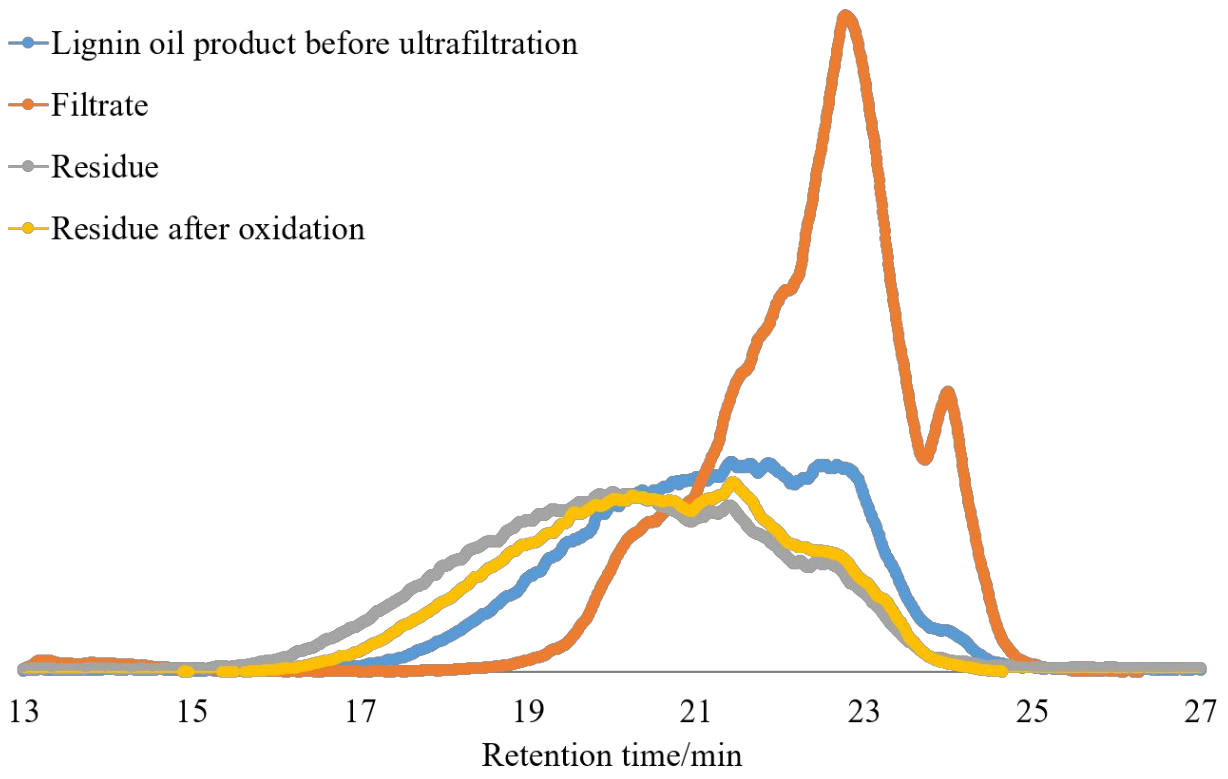

Figure S13. Characterization of the lignin oil products before and after ultrafiltration and the products after oxidation of residue by HPSEC, method B. The samples were from a 24 hours experiment in EtOH-H_2O (50/50, v/v) mixture catalyzed by 5% Ru/C under 20 bar H_2 and 240 °C. The oxidation experiment was conducted in EtOH-H_2O (50/50, v/v) mixture catalyzed by 5% Ru/C under 5 bar O_2 and 240 °C.
